# Super-resolution imaging reveals resistance to mass transfer in functionalized stationary phases

**DOI:** 10.1126/sciadv.ads0790

**Published:** 2025-02-14

**Authors:** Ricardo Monge Neria, Muhammad Zeeshan, Aman Kapoor, Tae Kyong John Kim, Nichole Hoven, Jeffrey S. Pigott, Burcu Gurkan, Christine E. Duval, Rachel A. Saylor, Lydia Kisley

**Affiliations:** ^1^ Department of Physics, Case Western Reserve University, Cleveland, OH 44106, USA.; ^2^ Department of Chemical and Biomolecular Engineering, Case Western Reserve University, Cleveland, OH 44106, USA.; ^3^ Swagelok Center for Surface Analysis of Materials, Case Western Reserve University, Cleveland, OH 44106, USA.; ^4^ Department of Chemistry and Biochemistry, Oberlin College, Oberlin, OH 44074, USA.; ^5^ Department of Chemistry, Case Western Reserve University, Cleveland, OH 44106, USA.

## Abstract

Chemical separations are costly in terms of energy, time, and money. Separation methods are optimized with inefficient trial-and-error approaches that lack insight into the molecular dynamics that lead to the success or failure of a separation and, hence, ways to improve the process. We perform super-resolution imaging of fluorescent analytes in five different commercial liquid chromatography materials. Unexpectedly, we observe that chemical functionalization can block more than 50% of the material’s porous interior, rendering it inaccessible to small-molecule analytes. Only in situ imaging unveils the inaccessibility when compared to the industry-accepted ex situ characterization methods. Selectively removing some of the functionalization with solvent restores pore access without substantially altering the single-molecule kinetics that underlie the separation and agree with bulk chromatography measurements. Our molecular results determine that commercial “fully porous” stationary phases are over-functionalized and provide an alternative avenue to characterize and direct separation material design from the bottom-up.

## Teaser

Nanoscale in situ imaging and chromatography show that chemical separations can be improved by reducing polymers that block pores.

## INTRODUCTION

**Fig. 1.**
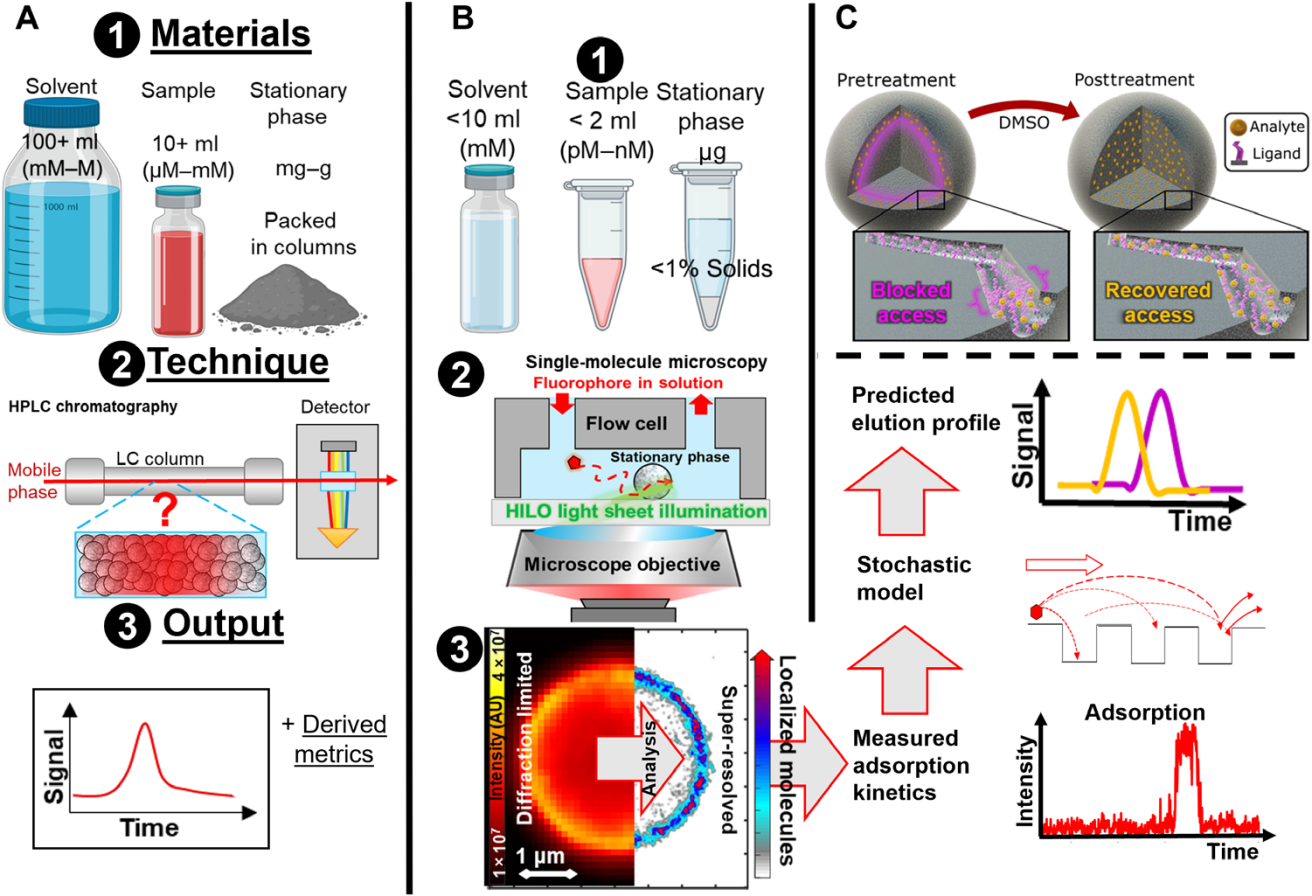
Single-molecule fluorescence microscopy directly observes the separation process to understand the performance of stationary phases using less materials and time. (**A**) The current paradigm in chromatography optimization relies on low-throughput, expensive, iterative trials. [(A), 1] High-performance liquid chromatography (HPLC) commonly uses of several milliliters to liters of solvent and sample, at high analyte greater than micromolar concentrations, and milligrams to grams of stationary phase material packed in columns both at analytical and preparatory scales. [(A), 2] HPLC data are collected as an ensemble measurement, typically using ultraviolet absorbance, after analytes pass through the column, causing all details of what occurs within the column to remain hidden. [(A), 3] The output is elution (signal versus time) curves from which all other metrics like theoretical plates and separation efficiency are derived. (**B**) Single-molecule microscopy measurements can predict the separation performance of a fully manufactured column with [(B), 1] less solvent, sample, and stationary phase. [(B), 2] Commercial porous stationary particles are deposited within microscopy flow cells. Analytes are introduced in solution and excited with a light sheet illumination and then imaged as they interact with the stationary phases. [(B), 3] The fluorescence signal is collected over space and time by a camera detector and subsequently improved to 30-nm super-resolutions using single-molecule localization analysis. Heterogeneous adsorption within stationary phases is directly visualized and the kinetics that drive the separation process are measured. Through stochastic model analysis, the kinetics are used to predict elution trends in a HPLC column experiment. AU, arbitrary units. (**C**) A cartoon representation of the mechanism single-molecule imaging reveals where dense functional groups block access to the inner volume of the porous functionalized particle, which are then removed by solvent treatment.

The separation of molecules from mixtures uses ~15% of the total energy in the US (*1*), emits 100 million tons of CO_2_, and costs $4 billion annually (*2*). For liquid chromatography, where an analyte in a liquid mobile phase differentially interacts with a solid stationary phase compared to other molecules, the current paradigm for optimizing the separation relies on low-throughput and expensive trials. Method development typically uses several centimeter-scale columns packed with grams of base material, passes liters of a multitude of mobile phase mixtures at varying pressures, and sacrifices moles of analyte (Fig. 1A). Hundreds or even thousands of combinations may be tested and eventually a working protocol may or may not be identified, but, critically, the fundamental factors that contribute to the separation (*3*) remain elusive in this trial-and-error approach.

Reliance on ex situ and ensemble-averaged techniques to characterize materials limits the physical understanding of what leads to a successful versus failed separation. Nitrogen adsorption isotherms measure gas uptake in milligrams to grams of stationary phase under non-hydrated conditions and low temperatures (e.g., 77 K) and use an assumed monolayer theory to extract surface area, pore volumes, and sizes (*4*–*6*). Imaging techniques, such as electron microscopy, provide nanoscale spatial measurements of pore sizes and morphology but require vacuum conditions and destructive cross sectioning (*6*, *7*). Analysis techniques like the method of moments, established by Hagel *et al*. (*8*) and Carta and Jungbauer (*9*), can extract information about chromatographic peaks by calculating their statistical moments (area, peak center, variance, and skew) to assess efficiency, broadening, and kinetic parameters (*10*–*12*). This approach can be paired with inverse size exclusion chromatography (ISEC) to infer porosity, accessibility, and dispersion indirectly under hydrated conditions by using a variety of inert tracers, like polystyrene standards, dextrans, polyethylene glycols, and glucose, and determine molecular weight distributions (*8*, *9*). However, the method of moments is very sensitive to noise, sampling, and approach for calculating the peak integral (*10*). Extracting molecular-scale behavior requires complex, often system/material-specific derivation and validation of mathematical models, and higher-order moments are notoriously difficult to interpret (*11*–*13*). Inferring kinetic information requires assumptions about parameters like complexation (*12*) and can fail to work with asymmetric profiles (*14*) that arise from heterogeneities in challenging separations (*15*). Similarly, ISEC itself averages behavior over ≥10^10^ stationary phase particles (*16*), assumes uniform pore shapes, and requires specific standard probes (*6*, *17*, *18*). Further, the relationship between retention volume in ISEC and pore size is notoriously complex (*8*, *9*).

In this work, we use in situ single-molecule super-resolution fluorescence microscopy of adsorption (*19*) for a molecular understanding and prediction of chromatographic performance with reduced amounts of materials and time. Here, we probe nanoscale adsorption using only micrograms of stationary phase, milliliters of solvent, and nanomolar concentrations of analyte (Fig. 1B). We use the exact same materials used in high-performance liquid chromatography (HPLC) contrary to previous research that used model thin film or isolated polymer samples (*20*) and non-functionalized silica (*21*) or focused only on diffusion, not adsorption, within zeolites (*22*). We visualize and spatially reveal steric hindrance limiting accessibility to the inner volume of chromatography particles and how access is recovered by decreasing the degree of functionalization (Fig. 1C). We demonstrate that microscopic data can predict HPLC elution profile trends and how super-resolution imaging can access molecular information to inform the design, development, and application of stationary phases (*23*).

## RESULTS

### Imaging adsorption within porous chromatography particles

**Fig. 2.**
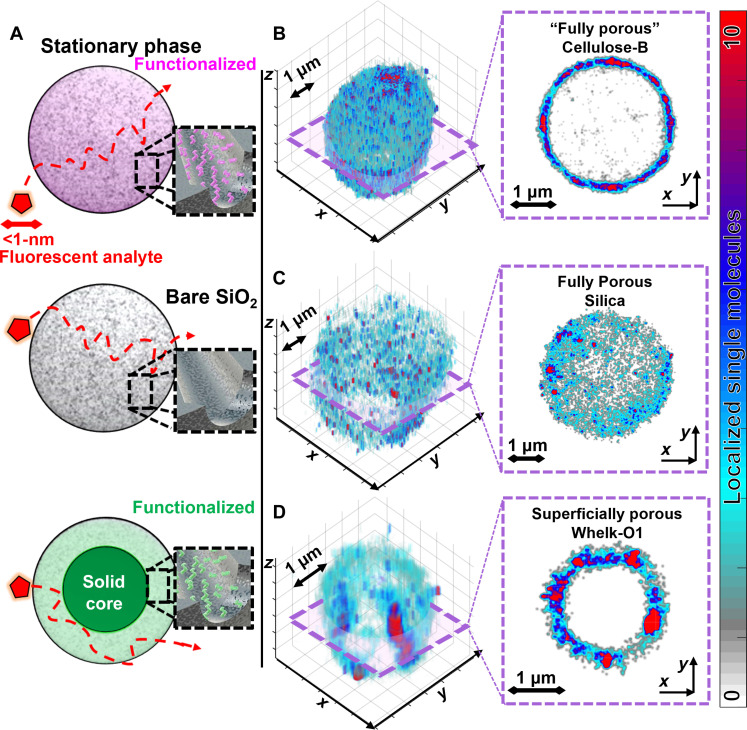
Super-resolution imaging reveals resistance to mass transfer in functionalized Cellulose-B chromatography particles. (**A**) We test the adsorption behavior of diffusing rhodamine 6G (red) fluorescent analyte in solution that interacts with different commercial stationary phase particles. We image 5-μm-diameter Cellulose-B functionalized (magenta) and bare non-functionalized silica particles (gray) FPPs, both with a distribution of ~100-nm mesopores, along with Whelk-O1–functionalized particles (green) with a superficially porous particle (SPP) design having a 0.5-μm-thick shell containing a distribution of ~10-nm pores and a 1.7-μm-diameter non-porous silica core. Analytes are expected to freely diffuse throughout the full volume of the FPPs but should be blocked from accessing the center of SPPs due to the solid core. See table S1 for further details. (**B** to **D**) Three-dimensional (3D) maps of single-molecule adsorption events collected throughout the ~10 to 65 μm^3^ volumes in 250- ± 10-nm axial steps, with example 2D slices at the half diameter for (B) Cellulose-B particles, (C) non-functionalized silica particles, and (D) Whelk-O1 SPPs. Heterogeneity is discerned by bright-red spots showing the most frequented adsorption sites. We find that access of analytes to the inner volume of the fully porous Cellulose-B is comparable to the SPPs, showing low accessibility to the pore network. Quoted fully porous is used to indicate difference between the marketed and our observed porosity.

We study stationary chromatography particles that vary in porosity and chemical functionalization (table S1 and Fig. 2A). Our main material of interest is Cellulose-B, which consists of 5- ± 0.8-μm-diameter (fig. S11) silica fully porous particles (FPPs), with a distribution of ~100-nm-wide mesopores, and functionalized with coated cellulose tris(3,5 dimethyl phenyl carbamate) (*24*, *25*). To understand the effect of functionalization, Cellulose-B is compared to FPPs of bare non-functionalized silica of the same porosity and size. We also image superficially porous particles (SPPs) composed of a 1.7-μm-diameter solid core surrounded by a 0.5-μm-thick, 10-nm porous silica shell, functionalized with 1-(3,5-dinitrobenzamido)-1,2,3,4,-tetrahydrophenanthrene (Whelk-O1). We further characterize FPPs with Whelk-O1 functionalization (fig. S3) and zwitterionic particles (figs. S1 and S4) to support our results. All subsequent analysis is normalized to account for differences in particles sizes.

**Fig. 3.**
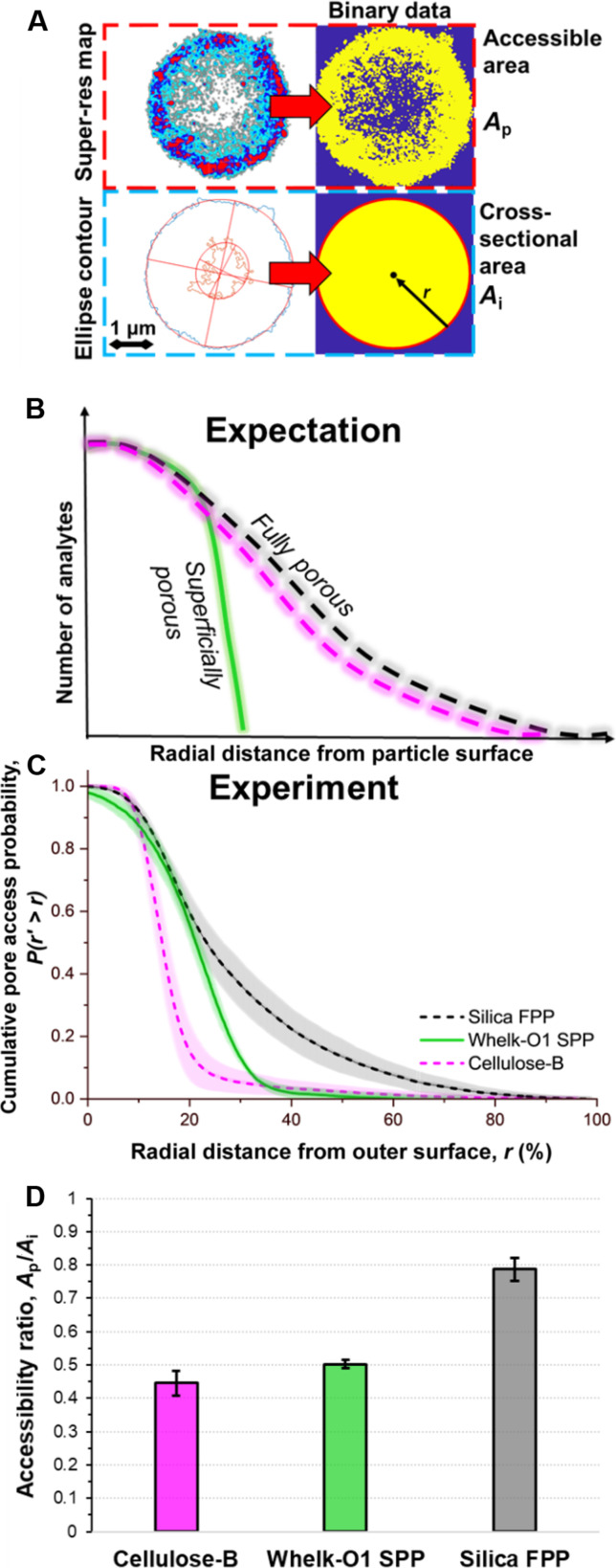
Single-molecule locations quantify spatial analyte pore accessibility. (**A**) The cross-sectional area (*A*_*i*_) and radius (*r*) of the imaged stationary phase particles is defined from fitting an ellipse to the binarized super-resolution map of all localized analytes. The area analytes can access (*A*_*p*_) is defined by the area covered by identified single molecules. The cumulative distribution of all identified single molecules as the distance from the outer edge of each particle, defined as a % of the particle radius *r*. (**B**) On the basis of particle designs, analytes are expected to freely access well into the volume of FPPs while reaching a sharp cutoff at the solid core for SPPs. (**C**) Experimentally, we find that functionalized FPPs restrict analyte access to the inner volume to a similar degree as functionalized SPPs. Data are average (line) and SD (shaded) from three different representative particles, constituting >15,000 single-molecule events per curve. Data on semi-log scale shown in fig. S5A. The relative accessible surface area from super-resolution imaging is calculated as the ratio of the area probed by analytes (*A*_*p*_) over the imaged cross-sectional area (*A*_*i*_) and plotted in (**D**). See section s9 for details.

We dynamically super-resolve the locations of individual fluorescent analyte molecules as they diffuse and adsorb within chromatography particles immobilized on coverslips (Fig. 1B, 2 and 3, and section s3). Rhodamine 6G is our model analyte: a fluorescent molecule with a measured hydrodynamic radius of 0.589 nm (*26*, *27*), comparable in size to molecules separated on Cellulose-B columns (*25*, *28*, *29*) (Fig. 2A). The analyte is introduced in a microfluidic flow cell at nanomolar concentrations and imaged with a highly inclined and laminated optical (HILO) light sheet with a thickness of 2.3 ± 0.5 μm (fig. S2) (*19*). The fluorescent signal of adsorbed single molecules is super-resolved (Fig. 1B3) by applying localization analysis (section s4) to map adsorption sites at 30- ± 20-nm lateral and 340- ± 50-nm axial resolutions that would be obscured in diffraction-limited images (Fig. 2, B to D) (*19*, *30*, *31*). The single-molecule observations provide adsorption kinetics data at 32-ms temporal elution to model elution (Fig. 1B3, right).

### The inner volume of functionalized porous particles is inaccessible to analytes

Super-resolution imaging reveals that analytes cannot freely diffuse throughout the volume of Cellulose-B and Whelk-O1 FPPs, contrary to the commonly accepted picture of diffusion throughout porous chromatography particles (Fig. 2B, and fig. S3). Conceptually, analytes are expected to undergo mass transfer throughout the volume of the Cellulose-B and silica FPPs (*32*) while being blocked by the solid core in Whelk-O1 SPPs (Fig. 2A). However, from super-resolution imaging, we find that the marketed “fully porous” functionalized stationary phases show very low accessibility to the inner porous particle volumes (Fig. 2B). All the stationary phases show nanoscale heterogeneity in adsorption, with discernable differences in affinity, where visually, highly frequented adsorption sites can be identified as the bright-red spots and weaker affinity as blue spots, respectively (Fig. 2, B to D). The super-resolution map of Cellulose-B is markedly similar to the Whelk-O1 SPP with a solid core (Fig. 2D) and does not resemble the silica FPPs (Fig. 2C), where analyte freely accesses the particle volume.

Quantifying the distribution of analytes within stationary phases contextualizes the unexpected pore inaccessibility. We generate cumulative distributions of the distance between single-molecule locations and the outer surface of the chromatography particles, defined as a percent of the particle radius (%*r*) (Fig. 3A). Analytes should fully probe the silica FPPs (Fig. 3B, gray) and functionalized FPPs (Fig. 3B, magenta), whereas the distribution should sharply drop for SPPs (Fig. 3B, green) at the %*r* distance defined by the porous layer thickness. However, our imaging shows sharp drops for both Cellulose-B and Whelk-O1 (Fig. 3C). Quantitatively, we define a limit of accessible distance into the particles, where 99% of analytes cannot reach (Fig. 3D). Experimentally, in silica FPPs, up to 84 ± 3%*r* is accessible, but only 48 ± 1%*r* for Whelk-O1 SPPs. The Whelk-O1 result is near the expected limit of ~37%*r* based on the ratio of the 0.5-μm-thick porous shell and the 1.35-μm particle radius. Comparably, for Cellulose-B 64 ± 2%*r* is accessible, equivalent to only a ~1.6-μm-thick porous layer for the 2.5-μm radius particles. Whelk-O1 FPPs (fig. S3D) and zwitterionic FPPs (fig. S4) showed similar core-shell–like spatial distributions.

### The observed superficial porosity is obscured in traditional ex situ characterization methods

Single-molecule spatial distributions quantify pore accessibility that is not discernable by other common characterization techniques. Nitrogen gas adsorption isotherms combined with BET (Brunauer-Emmett-Teller) analysis (section s10) are one of the most ubiquitous methods used to characterize porosity and surface area of chromatography materials (*4*). However, differences between the Cellulose-B and non-functionalized silica cannot be discerned by BET, both resulting in measured surface areas of ~40 ± 10 m^2^/g due to poor mesopore filling (see section s10), while Whelk-O1 SPPs measure a higher surface area of 138 ± 7 m^2^/g. Meanwhile, scanning electron microscopy (SEM) imaging of focused ion beam cross-sectioned Cellulose-B particles (section s11) shows the expected distribution of ~100-nm mesopores (fig. S10), and no physical signs of pore blocking. Yet, our imaging shows that these large inner pores are inaccessible to our small ~1-nm analyte in functionalized FPPs. Thus, for comparison to conventional methods, we quantify and take the ratio of the cross-sectional area probed by analytes (*A*_*p*_) per total imaged area of the stationary phase (*A*_*i*_) (Fig. 3A and section s9). Analytes only access 45 ± 4% and 50 ± 1% of the Cellulose-B and Whelk-O1 areas, respectively, compared to 79 ± 3% for silica (Fig. 3D). This provides a more direct quantification of the effectively accessible porosity in solvated conditions.

### Removing functionalized cellulose coating increases pore accessibility

We determine that the high degree of functionalization of the stationary phases leads to steric hindrance. We rule out our low operating pressure of ~700 psi by observing similar distributions at varying pressures (fig. S12). Using refractive index matching medium confirms that the functionalized material is not optically obscuring analytes (fig. S14). Changing pH conditions does not improve pore accessibility (section s21), and SEM imaging confirmed a large dispersion of ~100-nm pores (fig. S10), which should not size exclude the 0.589-nm fluorophore (*27*). The cationic rhodamine 6G dye attracts to the electrostatically negative silica and cellulose (*33*, *34*), which is further supported by repulsion of an anionic dye (section s22). The 20 mM buffers also favor adsorption by reducing cation competition (*24*). By testing the effect of removing the cellulose polysaccharide coating by treating the particles with 10, 20, and 100% (v/v) dimethyl sulfoxide (DMSO) in H_2_O (section s15), which is a strong solvent for cellulose (*35*), we show regained pore access.

**Fig. 4.**
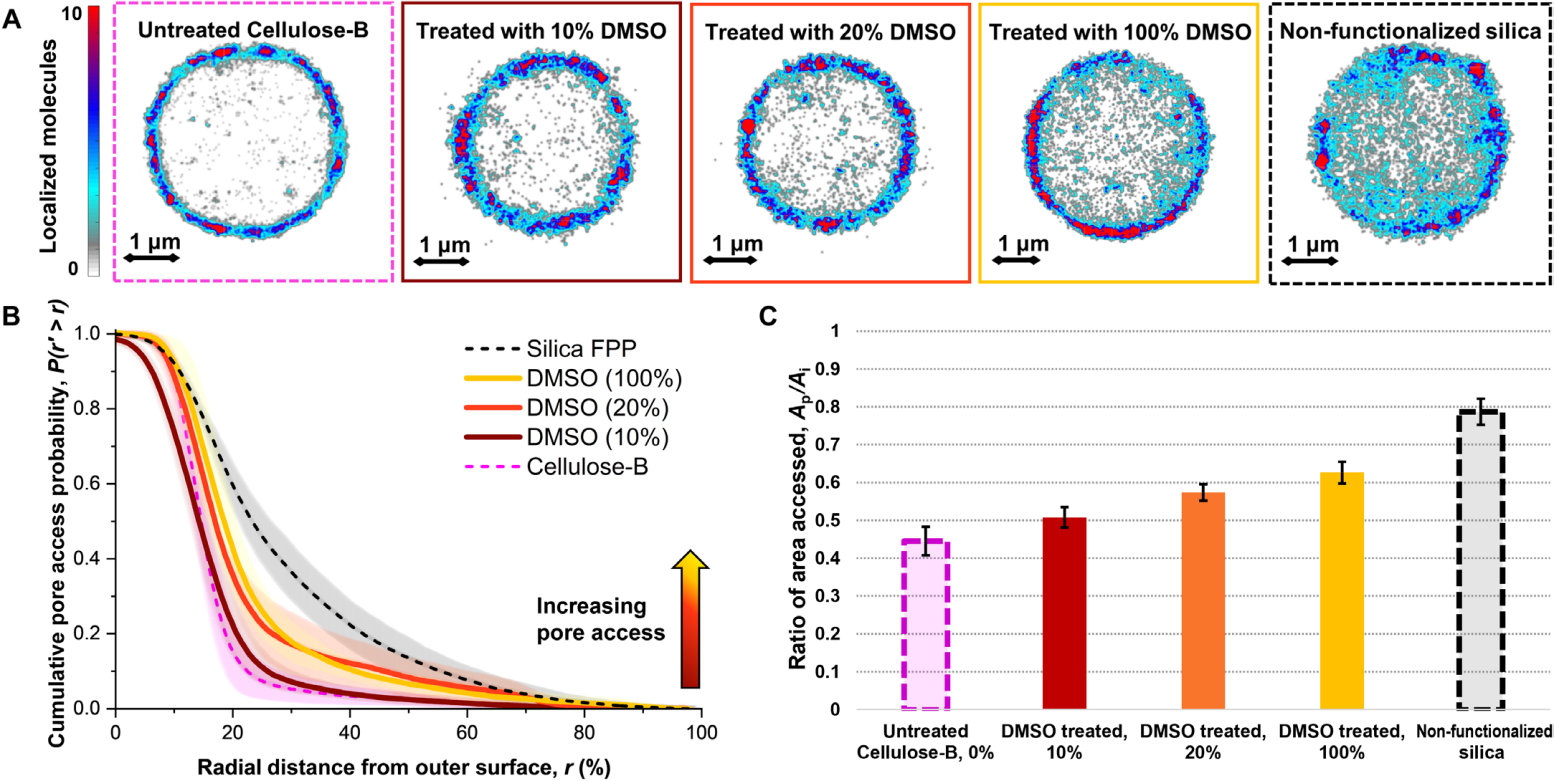
Pore network accessibility in Cellulose-B particles is increased by treatment with organic solvent. (**A**) Super-resolution maps show increased access to the inner volume of the particles after treatment in dimethyl sulfoxide (DMSO) solvent at varying concentrations. Cellulose-B particles were treated over 24 hours at 40°C. (**B**) The radial distribution quantifies the degree of recovery in pore access in the inner volume of the particles, approaching the original full pore volume of the silica gel backing as DMSO concentration is increased. Data are average (line) and SD (shaded) from three different particles each. Data on semi-log scale are shown in fig. S5B. Lack of full recovery shows that some loss in pore volume will occur with any coating present. (**C**) Accessible area by the analyte increases consistently with stronger DMSO treatment (i.e., decreasing degree of functionalization), showing a quantifiable measure of porosity in the same format as Fig. 3D.

We find that removal of the functionalized cellulose with increasing solvent strength increases pore accessibility in Cellulose-B (Fig. 4). Compared to the untreated Cellulose-B (Fig. 4A, magenta), samples treated with 10% DMSO (dark red) show a visible increase in analyte accessibility to the inner volume of the particles, with further improvements with 20% (orange) and 100% (yellow) solvent. Quantifying the spatial localizations, analyte accessibility inside particles increases with increasing solvent concentration (Fig. 4B). Notably, we successfully regain analyte access to the inside of Cellulose-B particles, reaching distances up to 86 ± 3%*r* (Fig. 4B, yellow) with 100% DMSO treatment, identical to the 84 ± 3%*r* observed for silica (Fig. 4C). Furthermore, we observe a consistent increase in accessible % surface area with increasing solvent strength, reaching up to 63 ± 3% in 100% DMSO, which remains less than the 79 ± 3% in silica (Fig. 4C).

Time-of-flight secondary ion mass spectrometry (ToF-SIMS) imaging corroborates the super-resolution microscopy results to determine that the functionalized polysaccharide coating blocks the pores in Cellulose-B. Elemental mapping of cross-sectioned particles reveals that the dense cellulose and chlorine-containing tris(3,5 dimethyl phenyl carbamate) groups are reduced primarily near the outer edges of the particles (fig. S16A). After treatment with 100% DMSO, ToF-SIMS shows the functionalization is only partially removed, leaving chlorine-containing groups throughout the particles (fig. S16B). We attribute the primary reason behind blocked pore accessibility in the Cellulose-B particles to a “shell” of functional groups forming near the outer surface of the particles, preventing analyte access to the inner volume (Fig. 1C). Particle pore accessibility is not fully recovered, likely due to the remaining functionalization after solvent treatment (fig. S16B). Improved solvent intrusion or application of other solvents could potentially better dissolve the cellulose than DMSO (*35*), but these solvent-based methods can further alter the surface chemistry and can be inconsistent. Rather than trying to optimize solvent-based removal, we would advise better control of the degree of functionalization in the manufacturing process during the deposition of the cellulose to the silica.

### Analyte adsorption kinetics that underlie separation reveal HPLC elution trends

**Fig. 5.**
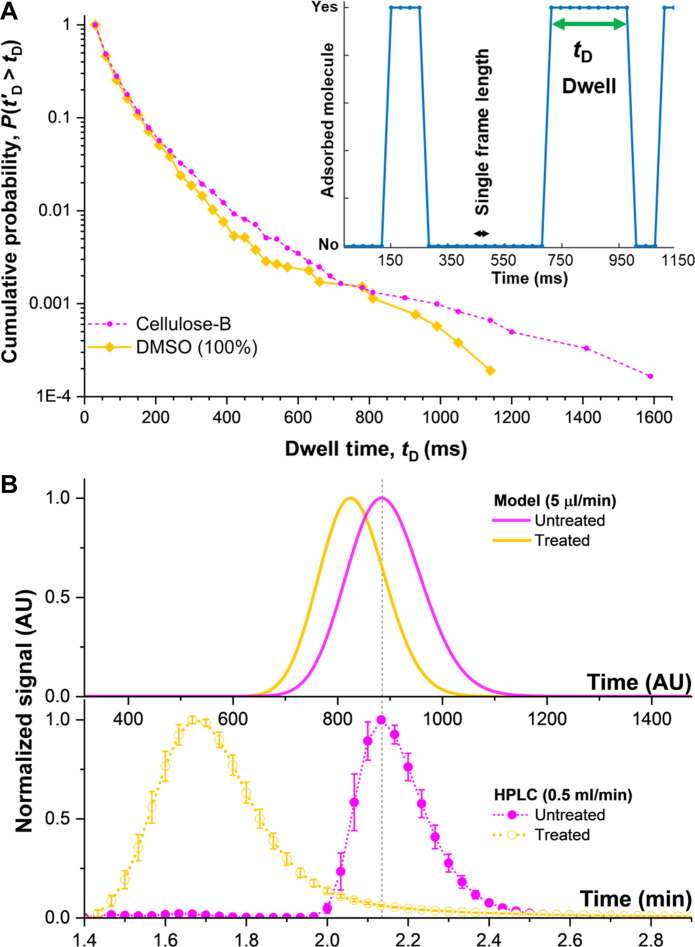
Single-molecule adsorption kinetics in porous stationary phase particles reveal differences in scaled chromatographic elution. (**A**) Inset: Single-molecule kinetics of adsorption are measured as the time analyte molecules spend stuck [dwell time (*t*_D_)] at individual nanoscale adsorption sites throughout the imaged chromatography particles. (A) Cumulative distributions of measured dwell times for thousands of single-molecule adsorptions observed over at least three chromatography particles of each type. (**B**) Top: Modeled elution from single molecule observations shows removal of the outer functional layer of Cellulose-B particles with DMSO treatment reduces overall retention time, while maintaining longer specific adsorptions for separation. Bottom: Experimental elution peaks of rhodamine 6G analyte, measured on commercial chromatography columns (REFLECT C-Cellulose B, 5 μm, 5 cm by 4.6 cm, Regis Technologies) on a HPLC system under comparable solvent and analyte conditions as in single-molecule measurements (see section s20). Both the model and experimental HPLC elution peaks show tailing characteristic of strong adsorptions, with faster elution (shift to the left) after DMSO treatment of Cellulose-B.

The temporal data in our single-molecule measurements allows us to predict ensemble chromatographic elution behavior (Fig. 1B3) as mass transfer and elution are dependent on the strength and duration of adsorption time. Our super-resolution maps (Fig. 2) include temporal information (Fig. 5A, inset) of the time individual molecules stay adsorbed on a site [dwell time (*t*_D_)] that are related to desorption rates (*k*_d_ = 1/*t*_D_), along with association data. Our results align with the stochastic theory of chromatography (*36*), where individual adsorption sites have first-order kinetics, but heterogeneity between different sites leads to an “*n-*site” distribution (*37*). Experimentally, we measure adsorption free energies varying from −2.9 to 11.4 kJ/mol, reasonable values for nonspecific adsorption of the rhodamine 6G analyte to the stationary phase surfaces (fig. S17) (*38*, *39*). Using the stochastic theory with the Lévy process representation (see section s19) (*40*, *41*) for all adsorption events within the particles (Fig. 5A), we model the effects of the heterogeneous kinetics on the anticipated ensemble elution profiles (Fig. 5B, top) (*40*, *41*).

Single-molecule adsorption kinetics show that DMSO treatment does not change peak shape notably while decreasing elution time (Fig. 5B, top). The overall single-molecule dwell times do not shift substantially after treating Cellulose-B particles with DMSO solvent (Fig. 5A, yellow), as the cumulative distribution curve shape remains close to that of untreated particles (Fig. 5A, magenta). This kinetic behavior further supports that functional groups remain even after DMSO treatment. There is a reduction in rare long-lasting adsorptions, which are associated with elution peak tailing and slower elution in a chromatography column. Our modeled curves reflect this change where the elution occurs earlier for DMSO treated particles (Fig. 5B, top).

Larger-scale, bulk HPLC separations using commercial packed chromatography columns validate and contextualize our model elution curves (section s20). The HPLC results show similar trends to our model for a Cellulose-B column before and after treatment with DMSO solvent (Fig. 5B and fig. S19), where elution time is faster and tailing due to strong adsorption (or resistance to mass transfer) is not changed considerably. While the relative degree of the temporal shift in bulk does not exactly match the predicted changes from the model elution, our single-molecule experiments successfully predicted the faster elution and general peak tailing shape (fig. S19) observed in the bulk separation only using dwell times. Band broadening in HPLC includes additional effects of column packing, Eddy, and longitudinal diffusion, which the current model does not account for, and differences in DMSO treatment approach (section s15 versus section s20) can cause different degrees of functionalization. Yet, the agreement in trends supports that accessibility and mass transfer are changed with DMSO treatment, leading to the faster elution.

## DISCUSSION

Our application of super-resolution imaging reveals several findings that impact liquid chromatography performance, chief among being pore occlusion due to polymer modifications. This phenomenon has thus far only been anecdotally discussed in the field and inferred from phenomenological theories applied to indirect measurements (*17*, *42*, *43*). Here, we directly probe and visualize limited pore accessibility in functionalized stationary phases, even for very small analytes (Fig. 2), and our experiments (Fig. 4 and figs. S10, S16, S20, and S22) confirm physical blockage, or rather steric hindrance, as the cause. We identify excessive ligands near the surface of particles (fig. S16) and find that their removal can speed up elution and decrease resistance to mass transfer (Fig. 5). These results provide further insight into the selectivity/permeability trade-offs often observed in bulk chromatography experiments, and the related challenges in ligand utilization and accessibility for resin-based purification of biologic analytes (*44*–*47*).

We further demonstrate that single-molecule mass transfer analysis can directly predict trends in HPLC elution profiles (Fig. 5). Prior single-molecule imaging of chromatography mainly used model surfaces mimicking stationary phases, solely reported single-molecule data (*21*, *23*), or required including unobservable kinetics (*48*) or construction of a simplified chromatography setup (*49*–*51*) to reach agreement between profiles. Here, we measure the same exact chromatography material on the microscope and within an HPLC column and observe similar trends between the two systems despite their vastly different scales (from micrometers to centimeters) (Fig. 5B). Simulated elution profiles from single-molecule measurements and experimental HPLC data both show faster elution and similar peak tailing after solvent-induced de-functionalization. These single-molecule observations identify and deconvolve the major role of mass transfer adsorption kinetics, rather than Eddy or longitudinal diffusion (as predominantly assumed), in a column-scale, bulk separation (*52*).

Mechanistic understanding of pore accessibility, mass transfer, and their effects on elution is not apparent with diffraction-limited imaging or ex situ methods. Past diffraction-limited scanning confocal microscopy produced static images that could not resolve adsorption sites or dynamics (*30*, *31*). While SEM images the porosity (fig. S10), it requires vacuum conditions without solvent or analytes of interest, ignores factors like pore swelling, and thus cannot discern pore accessibility. Similarly, nitrogen adsorption isotherms require dry conditions, and the small N_2_ probe can overestimate the accessible surface area (*53*, *54*). In our tests, these isotherms incorrectly reveal identical surface areas for silica and Cellulose-B (fig. S8) due to fundamental assumptions within the theory (section s10). Yet vendors list the BET surface area as a standard metric for some of the materials tested here (*55*). Comparably, in situ super-resolution fluorescence microscopy provides a direct, nondestructive measurement, with access to varied (size, charge, etc.) analytes, and tunable (pH, polarity, etc.) solvated conditions to match those used on columns. As accessible area, *A_p_*/*A_i_*, commonly dictates column size requirements, we propose that this metric, quantifiable via microscopy (Fig. 3D), will better inform the design of separation schemes.

Similarly, application of super-resolution imaging can directly probe the effects of stationary phase manufacturing methods. Measuring changes to analyte dynamics can efficiently provide engineers and commercial vendors clear information to design and optimize new separations more readily. Our imaging used only micrograms of stationary phase placed on microscope coverslips, milliliters of solvent, and nanomolar concentrations of analyte to measure mass transfer efficiency, a >1000-fold reduction in the resource load of traditional chromatography optimization. Mass transport and elution trends could be profiled with micrograms of material before committing to the scale-up required for columns. This could enable the testing of rare or newly synthesized materials and strategies, such as remote location/extreme environment synthesis and molecular foundries that simply cannot afford low-throughput separations.

We note some current limitations of super-resolution imaging. Imaging requires a photoluminescent signal from the probe, but fluorescent molecules can be strategically selected to mimic desired small-molecule analytes or bioconjugated to larger biologics. Our solvent treatment is not targeted, which can cause discrepancies between HPLC versus modeled elution speed and broadening, as degrees of functionalization might differ. Regardless, our kinetic results support that we maintain selectivity and similar adsorption after treatment, and both techniques agree in elution trends (Fig. 5). Here, we achieve linear flow velocities comparable to what is present in a column (section s24), and both literature (*43*) and our observed trends (section s12) suggest that pore accessibility decreases with increasing flow rate. However, our pressure range is limited to <1000 psi due to the substrate required for microscopy. We are presently working toward imaging at >30,000 psi using capillary-based approaches developed in biophysics (*56*).

The practical applicability for industry or others to use our super-resolution imaging approach for separations requires a single-molecule fluorescence microscope, sample preparation, and data analysis. Microscopes capable of single-molecule detection can range from ~$20,000 USD for a home-built setup (*57*, *58*) to multiple~$100,000’s for a multicolored, serviceable, plug-and-play commercial system. For the former, tutorials with open-source three-dimensional (3D) printable components have made performing imaging at the ultimate limit of detection of a single molecule accessible for nonexperts (*57*, *59*). Next, sample preparation requires little cost with the above-noted low material requirements. Our method (sections s2 to s4) uses 800 μg of stationary phase, the disposable flow cell components (<$2 per sample), and 1.2 ml of solvent for a typical experiment that generously takes ~4 hours for multiple samples. Last, the analysis after collection takes <10 min to 1 hour (depending on the dataset) on a desktop using our publicly shared analysis algorithm (see section s4) (*60*). Once fitting parameters for a set of data are determined, analysis of our typical experiments of three isolated particles, under three sample conditions (i.e., nine, 2000 frame movies, at 32 fps; see section s4), could be fully processed in ~20 min.

Improved “real-time” (*61*) analysis methods could further predict elutions in minutes. The large super-resolution biophysics community has developed rapid super-resolution algorithms for reconstructing cellular structures [e.g., RapidSTORM, QuickPALM, pSMLM-3D, and QC-STORM, in ImageJ/Fiji, among others; (*62*, *63*)]. The development of similar algorithms that include kinetic and elution profile analysis specifically for chromatography would further make super-resolution screening of chromatography an approachable technique for industry to adopt. Incorporation of GPU hardware and easy-to-use graphical user interface (GUI)-based environments have achieved analysis as fast as 3 × 10^6^ localizations per second and reconstruct super-resolution images from 10,000 frames of data in less than a minute (*64*). Development of chromatography-specific methodology is a subject of future work, and, as noted in our previous perspectives (*23*, *40*), we invite others from both the microscopy and the separation science communities to join in this pursuit.

Therefore, after the initial one-time microscope purchase, the cost and time to super-resolve analytes and predict elution profiles can be less than typical trial-and-error chromatographic screening. The physical information on single particles and single adsorption events and low material requirements makes our approach well suited for testing and development of new stationary phases that can be challenging to prepare in high yields. Even low-volume 1-ml columns used in process chromatography require grams of materials, which may be prohibitive for testing stationary phases prepared using new synthetic procedures or bio-based ligands such as recombinantly expressed proteins. Work toward imaging packed columns and higher pressures could extend the applicability of imaging to be relevant to chromatography development in the future.

Beyond this work, which imaged five different stationary phases spanning coated- and covalently bound functionalization, along with fully porous and superficially porous designs, the scope of materials with polymer or ligand-modified porous particles that can be studied with super-resolution imaging is expansive. This includes reverse-phase, normal-phase, hydrophobic interaction, chiral, and ion exchange chromatography that are ubiquitously used for multi-scale separations in (bio)pharma, energy and chemicals, environmental science, food and beverage production, among others. With Cellulose-B, ligand loading and functionalization strategies (*65*) that maintain pore access should be explored. Coupling these strategies with state-of-the-art or emerging stationary phase morphologies (monoliths or membrane adsorbers) (*66*) could further increase ligand accessibility; however, changes to stationary phase morphology may impact the surface area and capacity (*47*). Future work can aim to develop methods to quantify the Eddy diffusion in packed particle or membrane samples, increase operating pressures, and improve microscope spatiotemporal capabilities to better bridge the single-molecule and column scales.

## MATERIALS AND METHODS

An extensive description of the chemicals, microscopy, and analysis are fully detailed in sections s1 to s4, s10, s11, s17, and s20.

### Single-molecule sample preparation and microscopy

No. 1.5 microscopy slides were cleaned by submerging in a base peroxide (H_2_O_2_ + NH_4_OH + H_2_O, at a 1:1:6 volume ratio, respectively) bath at 70°C for 90 s, water rinsed, dried with nitrogen gas, then plasma cleaned in an O_2_ plasma cleaner at 140 to 280 torr, medium power, for 2 min. All uses of water in this work used type 1 ultrapure water. Different water suspensions of 0.1 wt % solids were used to deposit the stationary phases (table S1) on to the coverslips. An 8-μl volume of the stationary phase solutions was then dropcasted on the slides, where the cleaned surfaces were hydrophilic, allowing for physisorption by polar interactions with the 0.1% solids suspended in water (*19*). The slides were then covered by silicon flow cells and rinsed multiple times with water and then left to rehydrate for at least 2 hours before imaging. Single-molecule adsorption was characterized by HILO sheet fluorescence imaging of nanomolar concentrations of fluorescent rhodamine 6G dye under flow, as previously described (*19*) and detailed in section s3. Imaging was primarily done with 1 nM rhodamine 6G solutions in 20 mM 4-(2-hydroxyethyl)piperazine-1-ethane-sulfonic acid (Hepes) buffer (pH 7.33) flowed at a rate of 5 μl/min.

### Single-molecule localization and super-resolution image analysis

Single molecules were then identified and super-resolved using a home-written, publicly available (*60*, *67*) MATLAB (2022b) analysis. Further details can be found in (*19*) and section s4. From the resulting super-resolution images, we quantified the approximate area of each particle that was accessed by our rhodamine 6G analyte by binarizing the super resolution images in MATLAB (fig. S6B), producing a contour plot (fig. S6C), and estimating the total area with an ellipse (fig. S6D). Full details are included in section s9.

### Nitrogen adsorption isotherm measurements

Nitrogen adsorption isotherms for the chromatography particles were collected on a Micromeritics TriStar II 3020 physisorption analyzer. Samples of 150 mg of stationary phase were activated overnight at 150°C under continuous flow of N_2_. Adsorption-desorption isotherms were measured at −196°C, and the relative pressure range (*P/P*_o_) of 10^−6^ to 1 bar. Data in the partial pressure range of 0.05 to 0.3 were used in the BET eq. S1 (*4*) to estimate the surface areas of samples. The thermal stability of 10 mg of each sample was also assessed via thermogravimetric analysis (Discovery TGA 55, TA Instruments) as shown in fig S7. Full details are provided in section s10.

### SEM particle imaging

SEM images of Cellulose-B particles were collected using a Helios NanoLab 650 SEM using an accelerating voltage of 10 kV and probe current of 0.2 nA (fig. S11C). Approximately 5 mg of dry particles were mounted on an aluminum SEM stub using double-sided conductive copper tape. Particles were cross sectioned by milling with use of a focused Ga^+^ ion beam and sputter coated a ~34-nm layer of Pd, with complete details provided in section s11.

### TOF-SIMS elemental mapping

Unpacked Cellulose-B particles were immobilized within a conductive silver paste. Ion milling was done on a Gatan Model 693 Ilion+ Precision Cross-Section Ion Milling System, which includes a PIPS Cold Stage Controller at −40°C. The sample was ion milled in an Ar background for 60 min at an accelerating voltage of 5.5 keV.

Elemental mapping (fig. S16) was carried out on a Physical Electronics nanoTOF TRIFT V system, with a primary liquid metal ion gun set to ^69^Ga^+^, 30 kV, 1 nA direct current (DC). Acquisition was carried out in unbunched negative mode, with e-gun charge compensation, aperture size of 50 μm, and an image scan size of 30 μm. Ion count signal was collected for ~30 min at each sample area. The secondary electron detector option on the ToF-SIMS instrument was used to capture secondary electron images purely as a visual reference of sample morphology (fig. S16, black and white). Elemental mapping of cross-sectioned particles before (fig. S16A) and after DMSO treatment (fig. S16B) was done with ^35^[Cl]^−^, ^12^[C]^−^, ^28^[Si]^−^, and ^16^[O]^−^ being our primary reference peaks. Section s17 provides a complete description of methods and discussion of the results.

### HPLC bulk chromatography

Chromatography experiments were performed on a Shimadzu LC-20 AD Prominence liquid chromatography instrument. The control, untreated REFLECT C-Cellulose B (5 μm, 5-cm length by 4.6-mm diameter, Regis Technologies) column was rinsed with mobile phase [70% ethanol (EtOH):30% 20 mM Hepes] at 0.3 ml/min for at least 2 hours before experiments. The DMSO pretreated REFLECT C-Cellulose B (5 μm, 5-cm length by 4.6-mm diameter, Regis Technologies) column was pretreated with 20% DMSO:H_2_O at 30°C for >18 hours (see section s20 for full details of DMSO treatment) and then rinsed with mobile phase (70% EtOH:30% 20 mM Hepes) for 4 hours before experiments. Rhodamine 6G (3 μl, 100 μM in 20 mM Hepes buffer) was run on each column (*n* = 4 replicate injections) with an isocratic mobile phase of 70% EtOH:30% 20 mM Hepes, pH 7.33, with a flow rate of 0.5 ml/min, and a column temperature of 30°C. Detection was accomplished using a photodiode array over the range 200 to 600 nm at 5 Hz. The resulting chromatographic data at 530 nm with a 4-nm bandwidth were analyzed with Origin 2022. Full details are provided in section s20.

## Supplementary Materials

### The PDF file includes:

Supplementary Text, sections s1 to s25 Tables S1 and S2 Figs. S1 to S23 Legend for data S1 References
