# Supplementary material for: Super-resolution imaging reveals resistance to mass transfer in functionalized stationary phases
Source: Sci Adv. 2025 Feb 14;11(7):eads0790. doi: 10.1126/sciadv.ads0790 (PMC11827638; doi:10.1126/sciadv.ads0790)
Supplement: Supplementary file 1 — Supplementary Text, sections s1 to s25 Tables S1 and S2 Figs. S1 to S23 Legend for data S1 References [file sciadv.ads0790_sm.pdf]

Science Advances
AAAS

Supplementary Materials for

Super-resolution imaging reveals resistance to mass transfer in functionalized stationary phases

Ricardo Monge Neria *et al.*

Corresponding author: Lydia Kisley, lydia.kisley@case.edu

Sci. Adv. **11**, eads0790 (2025)
DOI: 10.1126/sciadv.ads0790

The PDF file includes:

Supplementary Text, sections S1 to S25
Tables S1 and S2
Figs. S1 to S23
Legend for data S1
References

Other Supplementary Material for this manuscript includes the following:

Data S1

s1. Commercial Chromatography Materials Used.

We studied stationary phase materials that vary in porosity and chemical functionalization as detailed in Table S1, with chemical structures provided in **fig. S1**.

Table S1. Physical properties of commercial stationary phase particles, as provided by the suppliers.

Stationary Phase	Base matrix	Nominal Pore diam. (nm)	Nominal Particle diam. (μm)	Functionalization	Vendor	Additional Detail
Cellulose-B	Fully-porous silica	100	5	Cellulose tris(3,5 dimethyl phenyl-carbamate)	Regis	…
Silica	Fully-porous silica	100	5	None	Glantreo	…
Whelk-O1 SPP	Superficially-porous silica	10	2.7	1-(3,5-Dinitrobenzamido)-1,2,3,4,-tetrahydrophenanthrene	Regis	1.7 μm solid core; 0.5 μm porous shell
Whelk-O1 FPP	Fully-porous silica	10	3.5	1-(3,5-Dinitrobenzamido)-1,2,3,4,-tetrahydrophenanthrene	Regis	…

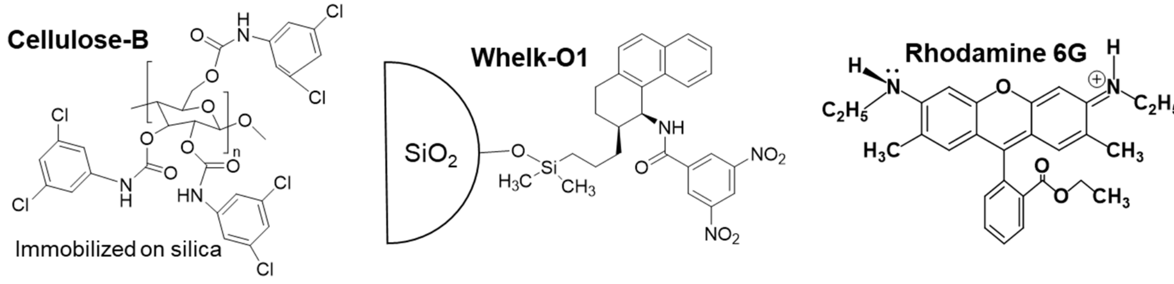

fig. S1. Chemical structures of tested stationary phase functionalizations and the fluorescent probe rhodamine 6G. Cellulose-B functionalization is coated on silica, immobilized by physisorption. Whelk-O1 ligands are covalently bound to the porous silica. Rhodamine 6G fluorescent dye has a partial cationic charge and a hydrodynamic radius of 0.589 nm (*27*).

s2. **Sample Preparation**
Commercial stationary phase particles were immobilized on silica glass for microscopy by relying on polar interactions, as previously described (*19*). Microscopy slides (20 mm x 30 mm, #1.5, Fisherbrand) were prepared by submerging in a base-peroxide (H_2O_2 + NH_4OH + H_2O, at a 1:1:6 volume ratio, respectively) bath at 70 °C for 90 s, water rinsed, dried with nitrogen gas (5.0 grade, Airgas), then plasma cleaned in an O_2 (Industrial grade, Airgas) plasma cleaner (PDC-32G, 115 V, Harrick Plasma) at 140-280 Torr, medium power, for 2 minutes. The base-peroxide bath was prepared with 30% Certified ACS, Thermo Scientific, H_2O_2, and Certified ACS Plus, Fisher Chemical NH_4OH. All uses of water in this work utilized Type 1 ultrapure water purified on an Elga Chorus 2+ system. Different water suspensions of 0.1 wt % solids were used to deposit cellulose tris (3,5-dimethylphenylcarbamate)-functionalized fully porous 5.0 μm silica stationary phase particles (Regis), non-functionalized 5.0 μm silica gel particles (SOLAS, Glantreo), 3.5 μm Whelk-O1 fully porous particles (Regis), and 2.7 μm Whelk-O1 superficially porous particles (Regis). The specific commercial particle parameters are summarized in Table S1. An 8 μL volume of the stationary phase solutions was then dropcast on the slides, where the cleaned surfaces were hydrophilic, allowing for physisorption by polar interactions with the 0.1% solids suspended in water. The slides were then covered by silicon flow cells (Hybriwell, 13 mm diameter, 0.15 mm depth, Grace Biolabs) and rinsed multiple times with water, then left to rehydrate for at least 2 hours before imaging.

s3. **Single-Molecule HILO Microscopy.**
Single-molecule adsorption was characterized by highly inclined and laminated optical sheet (HILO) fluorescence imaging of nanomolar concentrations of fluorescent rhodamine 6G dye (99%, Fisher) under flow, as previously described (*19*). The microscope body consists of an Olympus IX-73 inverted microscope, with a CNI diode laser (532 nm, MGL-III-100 mW) used for excitation. The laser light is guided through an achromatic doublet lens (visible, 50.8 mm, 400 nm EFL, Newport) mounted on a single axis translation stage (462 series, SM25, Newport) that allowed us to control the incident angle of the laser at the sample, set to a 77° exit angle to achieve HILO. An iris (Thorlabs, ID50/M 2.5 to 50.0 mm standard mounted iris diaphragm) is mounted before the doublet lens to restrict the illumination spot size, controlling the HILO sheet thickness. A *z* (vertical) single axis translation nano-positioner (Physik Instrumente, PIFOC, P-725) with a 400 μm travel range is paired with the 100x magnification oil immersion objective (Olympus, 100x, NA 1.49, UAPON100XOTIRF) to simultaneously control the imaging and illumination planes, allowing for 3D imaging. Imaging was done with 1 nM rhodamine 6G solutions in 20 mM HEPES buffer (Biotang Inc, pH 7.33), passed through the flow cells at a rate of 5 μL/min using a syringe pump (NE-1000, New Era Pump Systems Inc.). An EMMCD camera (iXon Life 897, Andor) collected the emission signal at 30 ms exposure, 400 EM gain, in Frame Transfer mode, with a ~ 2 ms readout time, and 532 nm laser excitation of 10 mW (379 W/cm^2) at the sample.

s4. **Single-Molecule Data Analysis**
Single molecules were identified and analyzed using a home-written, publicly available (*60*, *67*) MATLAB (2022b) code, run on a desktop (Intel(R) Core(TM) i7-8700; 3.20GHz CPU; 16.0 GB RAM). The scripts-package uses 2D Gaussian fitting strategies, paired with radial symmetry (*68*) centroid position refinements, to obtain super-resolved, single-molecule locations. We use the single axis nano-positioner to vary the vertical position of the imaging plane, first mapping the porous materials in 2D at each height, then stitching the planar slices as 3D voxels with a

modified version of Woodford's *vol3d_v2* algorithm (*69*). Further details can be found in Monge Neria and Kisley (*19*). For stationary phase imaging at a set *z* (i.e. vertical) position, we collect at least 2,000 frames (at ~ 32 frames per second). Overall, the sum of all observations for three representative particles used to generate a single curve (like in **Fig. 3**C) encompass on average > 15,000 total single-molecule events. This large number of events should be representative of the larger ensemble, and based on the ergodic hypothesis, their adsorption kinetics correspond to the ensemble average of the system at equilibrium (*70*, *71*).

In the super-resolution map reconstructions (like in **Fig. 2**) visually, high-affinity adsorption sites are represented by bright-red areas where >10 adsorption events occurred. Specific-adsorption sites are then further identified by satisfying two criteria: 1) The localizations of all the grouped events are within three standard deviations of the Gaussian PSF width and 2) their dwell time cumulative distributions are fit by a minimum of two-factor decaying exponential (see section **s18**). The cut-off threshold to differentiate specific and non-specific adsorption kinetics is defined by the capacity to reliably fit the dwell time distribution curve to a two-factor decaying exponential, where the first factor corresponds to random-nonspecific adsorption, and the second characterizes the rarer long-lasting specific adsorptions.

***s5.* Improving HILO Illumination.**

Signal-to-background is improved compared to our previous work (*19*) by the addition of an optical iris as a light stop on the illumination path, reducing the width and thickness of the light sheet. Similar to the characterizations detailed by Tokunaga et. al. (*72*), we measure the relative intensity ({signal – background} / {signal + background}) of fluorescent beads (Orange FluoSpheres, 0.2 µm diameter, 540/560 nm EX/EM, Invitrogen) immobilized by mixing within a 3% wt agarose (Sigma-Aldrich) hydrogel in water. The fluorescent beads were imaged under the same conditions as the single-molecule measurements of rhodamine 6G, with the 77° illumination exit angle to achieve HILO. An iris (Thorlabs, ID50/M 2.5 to 50.0 mm standard mounted iris diaphragm) placed right before the doublet lens was manually opened and closed to control the width of the illumination light sheet (**fig. S2**B-D), for which we achieve a minimum of 4.96 µm (**fig. S2**A). We record the average fluorescent intensity of the beads and background by scanning up in the z axis (−1 to 40 µm from the glass/gel interface) using the nano-positioner, then apply our single-molecule analysis algorithm. We define the width of the light sheet (*R*) by the full-width half-max of an illumination profile (**fig. S2**E), obtained by taking a linear cross-section of the intensity for the summed raw data over a full scan (**fig. S2**C) at different iris apertures. Note that field divergence causes higher intensity near the edges at larger iris apertures. Linear extrapolation of the sheet width vs. intensity profile was used to estimate the 116.5 µm max sheet width, based on the other measured profiles. From plotting the analyzed single molecule relative intensity data, we estimate the thickness of the light sheet (*dz*) as the estimated full width half-max of the data near the interface (**fig. S2**F). However, we do note that the use of a circular light-stop (rather than slits), and the use of a single area scan, does contribute to the noisier background signal and larger shifts in illumination seen at larger *R* widths (**fig. S2**F, insets). Finally, we plot out the measured light sheet thickness (*dz*) with respect to the measured width (*R*) of the profile and compare to the theoretical relation proposed by Tokunaga et. al. ($dz = R/\tan(\theta)$; θ =77°) (**fig. S2**G). From this we see that our light sheet matches the theory within error. We achieve a 2.3 ± 0.5 µm light sheet thickness for the operating ~11.36 µm width used for data collection when imaging stationary phase particles.

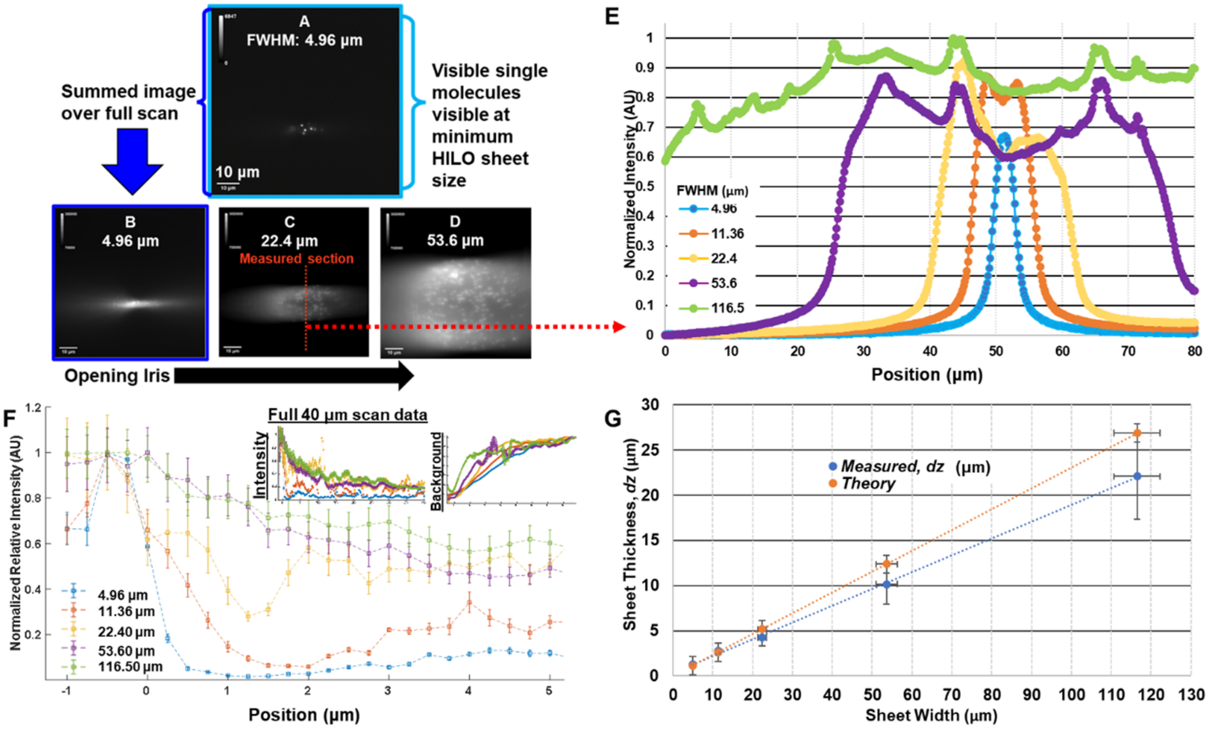

fig. S2. Imaging immobilized beads in agarose gel for HILO illumination characterization. (**A**) Fluorescent beads immobilized in agarose gel, imaged near the glass/gel interface at the minimum iris aperture, which results in a light sheet width (illumination area) 4.96 µm across. (**B**-**D**) Summed images of bead intensity raw data over a full 41 µm vertical scan through the gel sample, at varying iris aperture settings (i.e. varying sheet widths). (**E**) Width of illumination (*R*) was estimated by the width of the cross-sectional profile of the summed images, as shown in **C**. (**F**) Sheet thickness (*dz*) was approximated by the full width, half-max (FWHM) of the bead relative intensity profiles near the interface. (**F**, insets) Zoomed out full scan relative intensity profile, and the increasing background away from the interface. (**G**) Our measured sheet thickness (*dz*) vs. width (*R*) compared to the theoretical $dz = R/\tan(\theta)$, where θ = 77°. Note that the FWHM for incomplete profiles was estimated by doubling the half-width measured.

***s6.* Single-Molecule Results for Whelk-O1 FPPs**

Just like the rest of the data displayed in the main text, we collected single-molecule scans of fully porous Whelk-O1 (Regis) particles using our rhodamine 6G probes. The 3D super-resolution mapping (**fig. S3**A) shows similar spherical structure to all other imaged particles, but a lower overall number of adsorbed molecules, which visually results in a more grayish-cyan map, with very few distinguishably strong adsorption sites (red). We can partially attribute this to the smaller particle size (3.5 μm), and the possibility of some size-exclusion behavior occurring with the nominal 10 nm wide pores. Notably, when looking at a 2D slice (**fig. S3**B) we observe a very similar spatial distribution of localized molecules to those observed in Cellulose-B fully porous particles (**Fig. 2**), and quantitatively similar accessibility to the Whelk-O1 SPP and Cellulose-B particles (**fig. S3**C). However, upon further quantification of these distributions (**fig. S3**D), it becomes evident that this behavior more closely matches that of Cellulose-B particles treated with 100% DMSO solution. This suggests that this loss in pore accessibility could be attributed to pore filling caused by functionalization, instead of primarily a dense “shell” of functional groups near the particle surface (as we have seen with the Cellulose-B particles). The Whelk-O1 FPPs have smaller pores that are chemically functionalized with a smaller ligand than the large polysaccharide. Therefore, these results show any type of functionalization can result in loss of porosity and can strikingly lead to superficially porous behavior in “fully porous” particles.

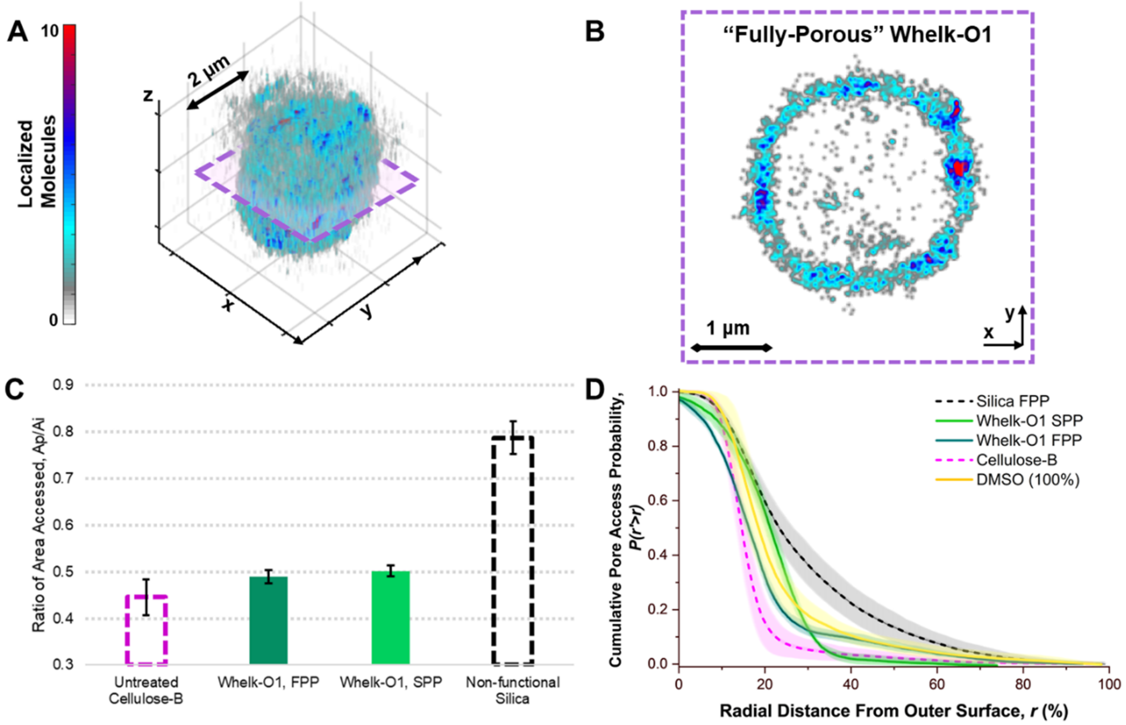

fig. S3. Single-molecule fluorescence characterization of Whelk-O1 FPP particles shows pore blocking behavior comparable to a Cellulose-B FPP. (**A**) 3D super-resolution map of single molecule adsorption events on a 3.5 μm Whelk-O1 fully porous particle (FPP), generated from 2D slices, scanning up in 250 nm steps. (**B**) 2D slice of single molecule adsorption at the approximate half-height of each particle. Access of analyte at the inner volume of the fully porous stationary phase is comparable to the Whelk-O1 SPP and Cellulose-B (**Fig. 2**), showing limited accessibility to the porous network. (**C**) The relative accessible area is estimated as the ratio of the area probed by single molecules (A_p) over the imaged cross-sectional area (A_i), showing very similar results to Whelk-O1 SPPs and Cellulose-B. (**D**) The distribution of analyte molecule adsorption radially from the edge of imaged particles shows very similar "core-shell" cut-off between all functionalized particles, but a gradual decrease on fully porous non-functional silica. The overlap between Whelk-O1 FPPs and Cellulose-B particles treated with 100% DMSO solution support our conclusion that an outer shell of functional groups was blocking the Cellulose-B particles, but the presence of any degree of functionalization will result in loss of porosity.

_s7._ Single-Molecule Results for Zwitterionic Particles
To strengthen our results, we have included data for zwitterionic (fully porous) particles from a different vendor (Chiral Technologies), demonstrating the pore accessibility is also limited in these stationary phases. We deposit Zwix(-) particles (3 µm diameter, ~10 nm pore size) and image following the same procedures detailed for the other tested stationary phases. Similarly, we flow buffered (HEPES, 20 mM, pH 7.33), 1 nM rhodamine 6G solutions and localized the single-molecule adsorption events over time. As shown in **fig. S4**A, the distribution of analytes remains primarily around the outside surface of the particles, similarly to what was observed for other tested functionalized resins. We observe that the analyte radial distance distribution function (**fig. S4**B) is fairly similar to the Cellulose-B functionalized, fully-porous particles. However, we note that we do observe much stronger binding of rhodamine 6G to the zwitterionic material than for Cellulose-B (**fig. S4**B, inset graph).

Overall, the variety of particle designs tested in this work represent a range of materials used in chromatography. We presented results for both coated (Cellulose-B) and covalently (Whelk-O1) bound functionalization, fully porous (bare silica, Cellulose-B, and Whelk-O1, zwitterionic) and superficially porous designs (Whelk-O1, zwitterionic), and the results of reducing functionalization of coated (DMSO treated Cellulose-B) particles. We demonstrated that both the coated and covalently bound functionalized fully porous particles show limited accessibility to the inner pores (**fig. S4**). Including zwitterionic particles, we present results for five different stationary phases from three different vendors.

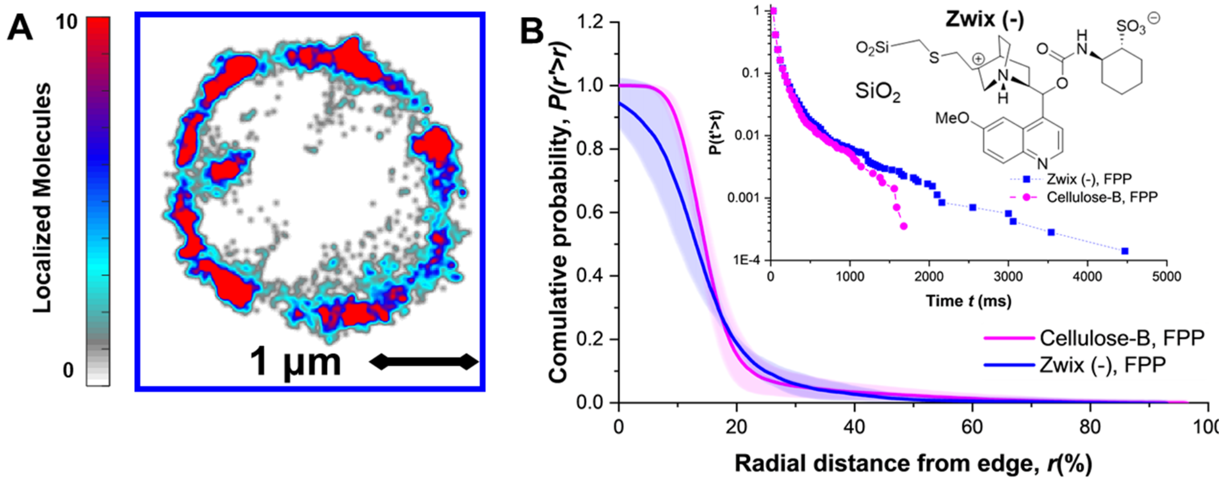

fig. S4. Fully porous zwitterionic particles, Zwix(-), show very similar analyte accessibility distribution to the functionalized Cellulose-B particles. (**A**) The single-molecule localization super-resolution map shows high adsorption of rhodamine 6G analytes, but low accessibility to the inner volume of the particles. (**B**) The distribution of analytes shows high similarity to the behavior observed on functionalized Cellulose-B FPPs. (**B**, insets) The zwitterionic functionalization shows stronger affinity for the analyte than Cellulose-B functionalization.

_s8._ Experimental Data in Semi-Log Scale

Alternative presentation of the single-molecule, cumulative spatial distributions as distance from the edge of measured chromatography particles presented in **Fig. 2-Fig. 4** in the main text. The distributions are presented with a log10 y-axis scale for better readability of the 0.01 (99% of analytes) axis cut-off discussed in the main text. The other point of distinction here is that the differences between the hard cut-off on the Whelk-O1 SPPs and the other FPPs is much more apparent, as seen by the sharp drop on the green curve in **fig. S5**A at ~61%r. The tail end of the averaged cumulative distributions can increase in variance and drop sharply due to the rarity (i.e. sparse data points) of molecules reaching deep into a particle volume. Results for DMSO treated particles are also included here in **fig. S5**B for comparison.

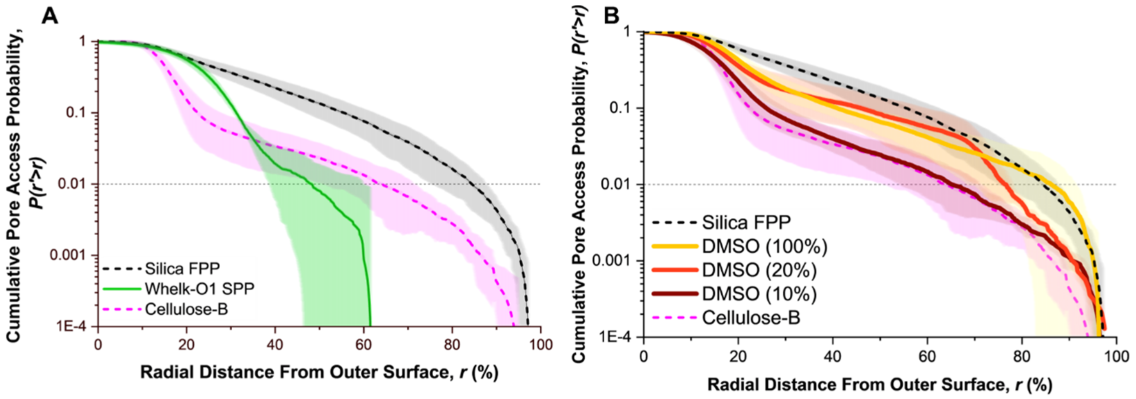

fig. S5. Single-molecule fluorescence characterization of chromatography particles shows pore blocking behavior. The log-linear cumulative distribution of all identified single molecules as distance from the approximated circumference of each particle, as a percent of each particle's estimated radius. Each line is generated from the average of 3 different imaged particles, with shaded regions corresponding to standard deviation. Semi-log plots of (**A**) data presented in **Fig. 2**, and (**B**) **Fig. 4** in the main text.

_s9._ Stationary Phase Imaged Area vs. Probed Area Approximations
In order to quantify the approximate area of each particle that was successfully accessed by our rhodamine 6G analyte, we estimate the total particle surface area in view based on the super-resolution images. First, a super-resolution map (**fig. S6**A) is generated by plotting every single-molecule localization as a simulated 2D Gaussian, with a width defined as the ~25 nm estimated specific molecule position-uncertainty. The super-resolution map is then binarized using Matlab's *imbinarize* function (**fig. S6**B), and the area probed by single molecules (A_p) is estimated using the *bwarea* function. A contour plot of the binarized map is generated using the *imcontour* function (**fig. S6**C). The contour data is extracted, then fitted to ellipses using an edited version of Gal's *fit_ellipse* function (**fig. S6**D).(*73*) Finally, for consistency, the cross-sectional area imaged (A_i) is estimated as the area of the fitted ellipse. The ratio of A_p/A_i is then used to generate the data used for Figures 3-4, **fig. S3**.

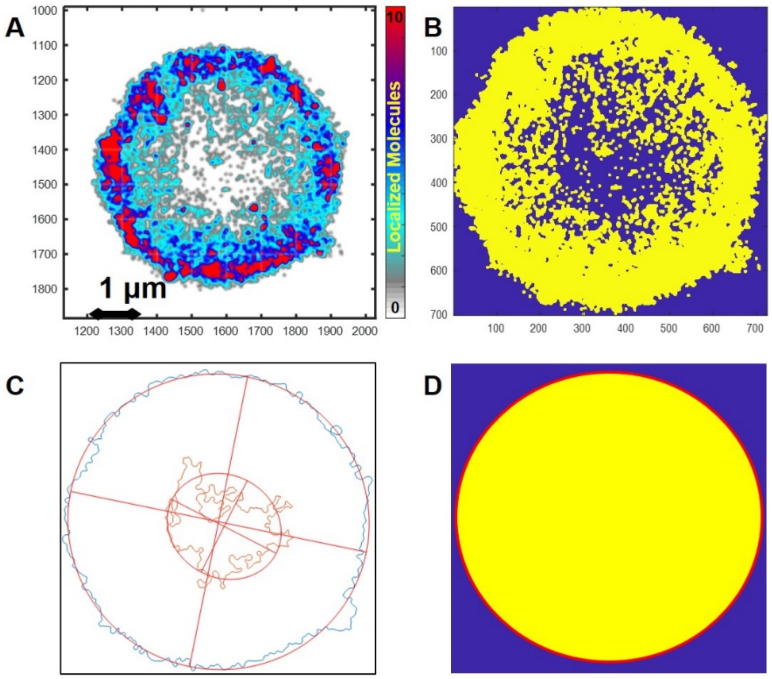

fig. S6. The total particle area imaged, Ai, and the approximate area probed by the R6G molecules, Ap, are calculated by generating custom super-resolution maps and fitting ellipses to their binarized contours. (**A**) The super-resolution map of a single imaged particle is generated from all the localized single rhodamine 6G probe molecules observed over 2000+ frames of collection. (**B**) The super-resolution map is zoomed into and binarized, from which the area probed (A_p) is estimated, then a contour plot is created from the binary image (**C**). The data from the contour is extracted, then fitted to ellipses using Gal's *fit_ellipse* function.(*73*) (**D**) The area of the larger outer ellipse is used as the estimate of A_i, the area imaged. Axis labels are in units of digital pixels, with each pixel corresponding to 8 nm in the physical sample.

_s10._ Nitrogen Adsorption Isotherms

Nitrogen adsorption isotherms for silica FPPs, Whelk-O1 SPPs, and Cellulose-B chromatography particles were collected. To examine thermal stability of the samples and ensure the absence of volatiles prior to nitrogen adsorption measurements, thermogravimetric analysis (TGA) was performed (TA instruments, Discovery TGA 55) by employing a ramp rate of 2 °C/minute from 25 °C to 500 °C under inert N_2 (5.0 grade, Airgas) atmosphere. Approximately 10 mg of a sample was placed in a platinum pan for each experiment. The obtained TGA curves of the samples are presented in **fig S7**.

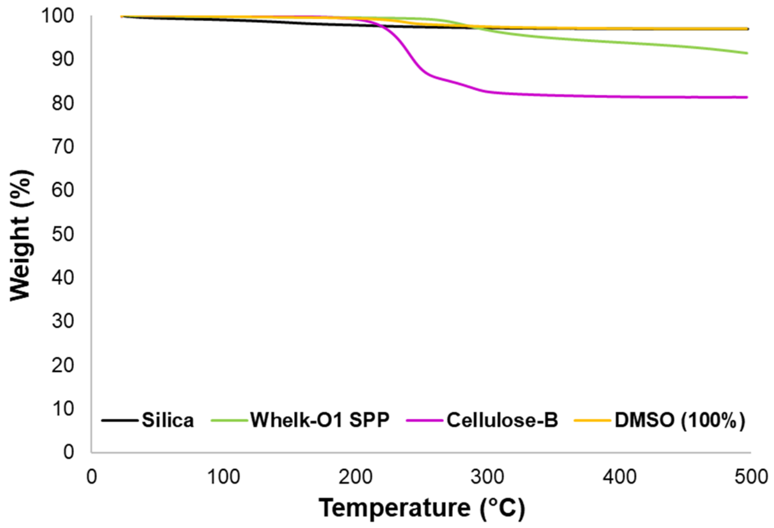

fig. S7. TGA curve of the sample under N_2 atmosphere up to 500 °C, demonstrating good thermal stabilities.

A Micromeritics TriStar II 3020 physisorption analyzer was used to determine the surface area of the studied samples through the Brunauer–Emmet–Teller (BET) analysis. Prior to each measurement, the samples were activated overnight at 150 °C under continuous flow of N_2 to ensure the removal of volatile residuals from the pores. Following the activation, N_2 adsorption–desorption isotherms were measured at -196 °C and the relative pressure range (P/P_o) of 10^{-6} – 1 bar. For each analysis, approximately 150 mg of sample was used. The isotherm data in the partial pressure range of 0.05 to 0.3 were used in the BET Eq. (S1) (*4*) to estimate the surface areas of samples. Results (**fig. S8**) were reproducible within an experimental error range of ±10%.

$$V/V_m = C(P/P_o) \,/\, [1 + \ (C - 1)\ (P/P_o)[1 - P/P_o)] \qquad \text{Eq. (S1)}$$

V/V_m represents the ratio of the volume of gas adsorbed (V) to the monolayer capacity (V_m), which is the volume of gas required to form a complete monolayer on the materials surface. C is the BET constant, which is related to the energy of adsorption in the first adsorbed layer and indicates magnitude of the adsorbent-adsorbate interactions. P/P_o is the relative pressure of the gas, where P is the equilibrium pressure of the gas at a specific relative pressure, and P_o is the saturation pressure of the gas. Data collection and analysis were carried out in duplicate, then averaged and error propagated to obtain the surface area measurements reported in **fig. S8**B.

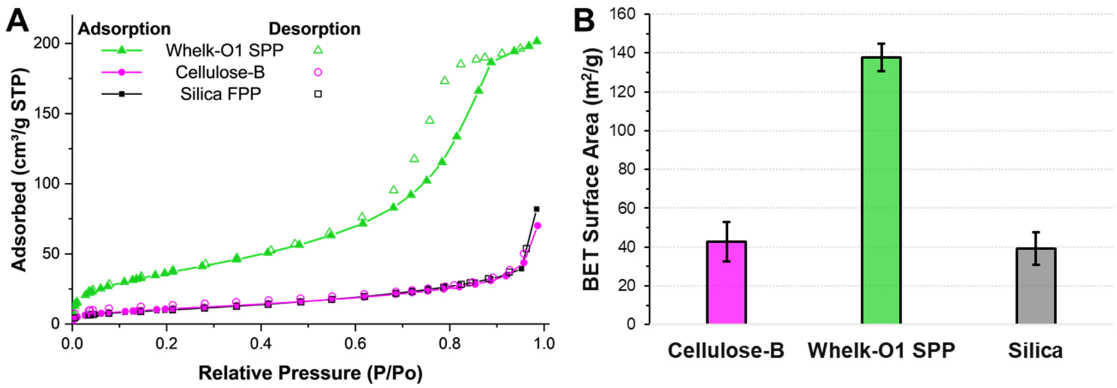

fig. S8. Nitrogen adsorption isotherms and BET surface area of chromatography particles. (A) Nitrogen adsorption-desorption isotherms obtained at -196 °C. (B) The surface area was extracted from N_2 isotherms using the BET method. The changes in accessible surface area for analyte adsorption revealed by *in situ* fluorescence in B are not resolved by the *ex situ* isotherms in D.

We find that the isotherm results do not resolve any difference between the mesoporous FPPs, with Cellulose-B and silica resulting in identical surface areas of 40 ± 10 m^2/g, matching the value reported as a specification by the manufacturer of the silica particles (*55*). However, the shapes of the isotherm curves are indicative of a non-adsorbing material. In contrast, the micro-porous Whelk-O1 SPPs have a characteristic “S” shape isotherm that corresponds to monolayer adsorption, resulting in 138 ± 7 m^2/g surface area (*74*). While nitrogen adsorption isotherms with BET analysis remains widely used (*75*), the theory relies on fundamental assumptions such gas monolayer formation as the first adsorption layer (*76*). Inspection of the Cellulose-B and silica isotherms (**fig. S8**A) exhibit negligible adsorption, indicating the measured surface area is due to weak adsorbate-adsorbent interactions, rather than adsorption at surface sites. The sharp increase in adsorption at high partial pressures could be related to progressive multilayer formation from N_2-N_2 interactions, and so the assumption for BET analysis is not fulfilled (*53*). Therefore, the nitrogen adsorption isotherms incorrectly reveal identical surface areas for silica and Cellulose-B. We also observed little to no difference in measured isotherm data between the Cellulose-B samples before and after treatment in 100% DMSO solution (**fig. S9)**.

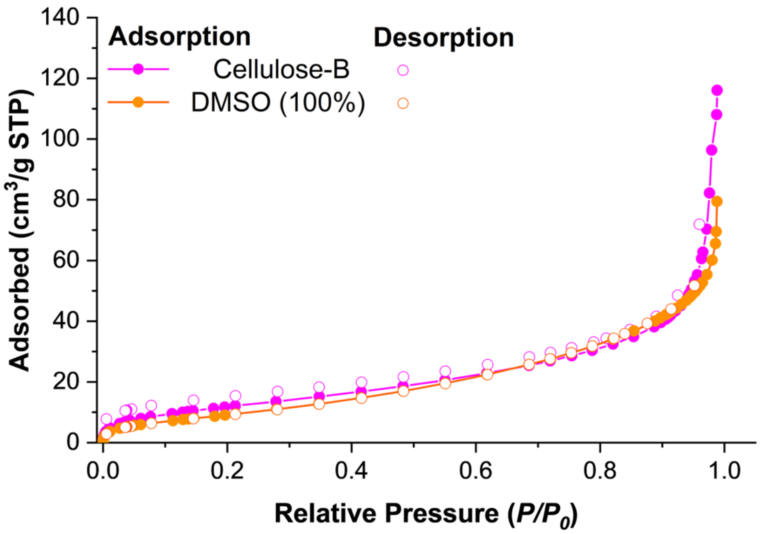

fig. S9. Nitrogen adsorption isotherms of Cellulose-B chromatography particles before and after treatment in 100% DMSO solution show little to no change in measured adsorption.

***s11.* SEM Particle Characterization and Size Distribution**

Scanning electron microscope (SEM) images of immobilized particles were collected using a Helios NanoLab 650 SEM. A small ~5 mg sample of dry particles was mounted on an aluminum SEM stub (1/2” slotted head, 1/8” pin, Ted Pella) using double-sided conductive copper tape (12.7 mm width, 3M^{TM}). The particles were deposited on the copper tape using a stainless-steel spatula, pressed lightly to ensure they adhered then the stub was tapped lightly at an angle to remove excess particles. The sample was prepared for SEM imaging and focused ion beam (FIB) milling by sputter coating a ~34 nm layer of Pd (Palladium Target, 99.95% Pd, Ø60 mm x 0.1 mm, from Ted Pella) using a Denton Vacuum Desk V turbo-pumped sputter coater. Secondary-electron images of single-layered particles on the tape surface (**fig. S11**) were acquired on a FEI Helios NanoLab 650 dual-beam SEM/FIB using an accelerating voltage of 10 kV and probe current of 0.2 nA (**fig. S11**C).

A cross-section of a single particle (**fig. S10**A) was milled using the FIB on the Helios NanoLab 650. Before milling, a protective cap of Pt was deposited on top of the single particle to reduce surface damage from the ion beam. (see top of **fig. S10**A). The Pt protective cap was deposited using an FEI Pt Deposition (CH3)3Pt(CpCH3) Gas Injection System. The Pt layer was deposited over an area of 7 µm (X) x 2 µm (Y), and 1.5 µm (Z) at ion beam conditions of 30 kV and 80 pA for ~9 minutes. Both milling and Pt deposition were enabled by the use of the focused Ga^+ ion beam. The first bulk milling was done using a regular cross-section at 30 kV and 0.79 nA for ~5 minutes over an area of 10 µm (X) x 10 µm (Y) x 5 µm (Z). The bulk milling was followed by 3 polishing steps using cleaning cross-sections at 30 kV and 80 pA for ~5 minutes each over an area of 10 µm (X) x 1.5 µm (Y) x 5 µm (Z). As shown with a linear section (**fig. S10**B), the nominal dispersion of ~100+ nm mesopore widths can be measured / observed from imaging of the particle interior.

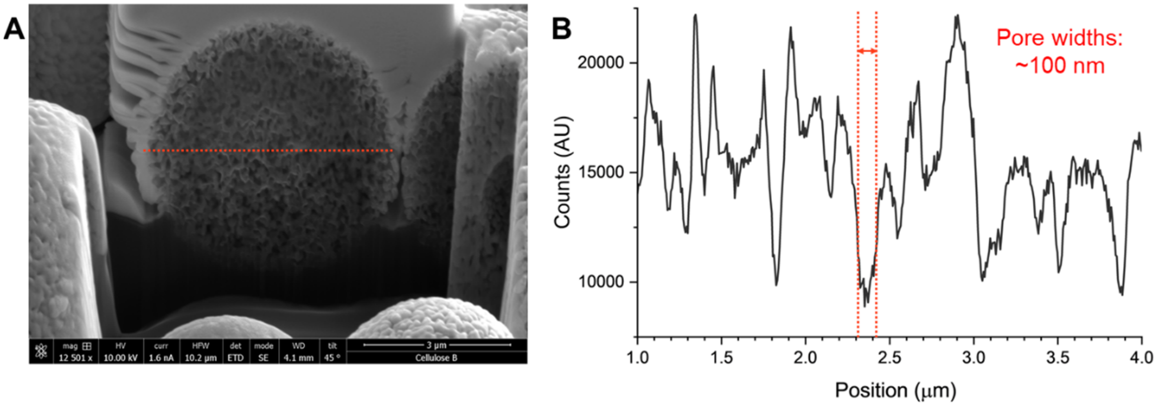

fig. S10. Scanning electron microscope (SEM) image of a cross-section of Regis Cellulose-B commercial chromatography stationary phases shows fully 100 nm porous structure. (**A**) A cross section of a single chromatography particle. Sectioning achieved by focused ion beam milling after depositing a Pt cap on top of the particle. (**B**) The pore structure is discernable and pore sizes measurable.

The high-resolution SEM images provide a good basis for measuring particle sizes, allowing us to generate a particle size distribution (**fig. S11**D). A SEM capture showing a large population of particles was selected (**fig. S11**C), then the size of each particle was approximated as an ellipse using ImageJ's analysis function 'Analyze Particles' after thresholding (*77*). From the obtained individual particle area measurements, the estimated diameters were simply calculated by approximating each particle area as a circle. The resulting distribution shows a mean particle diameter of 5.0 ± 0.8 µm, matching the vendor's reported nominal value of 5 µm.

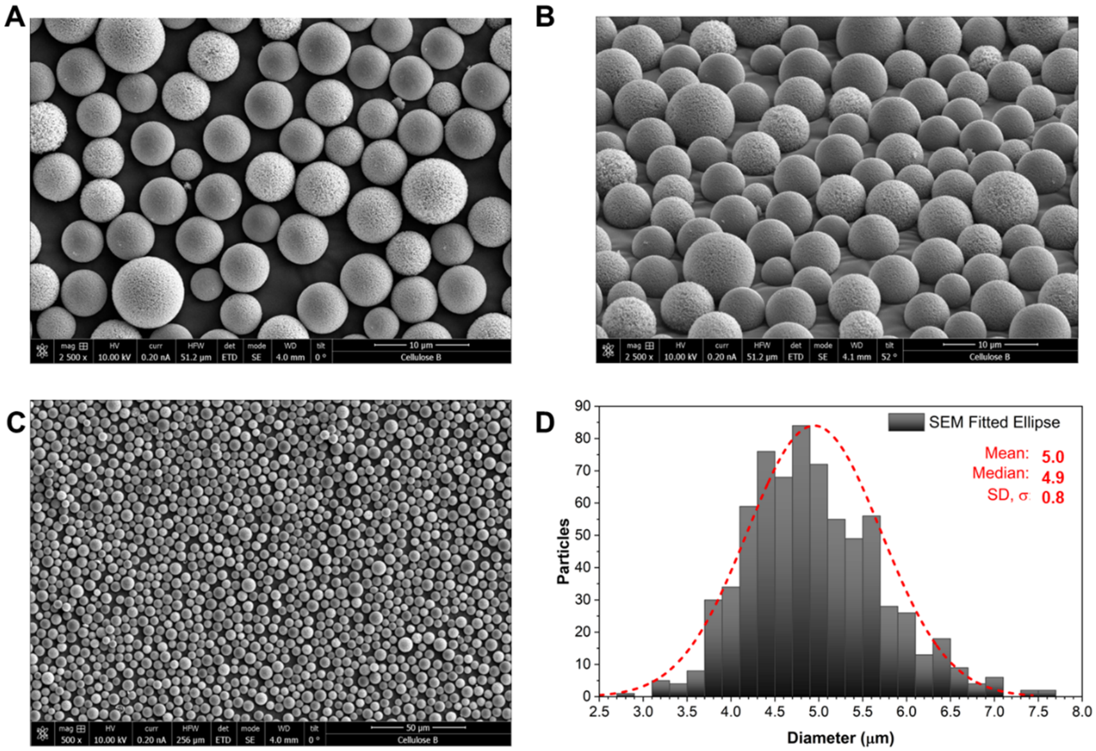

fig. S11. Scanning electron microscope (SEM) images of Regis Cellulose-B commercial chromatography stationary phases. (**A**) A top-down view of chromatography particles, where the surface pore structure is discernable and pore sizes measurable. (**B**) View at 52° tilt of the same particles shows further detail and morphology and visualizes differences in particle sizes. (**C**) Image of a higher density of single particles allows for estimation of overall particle sizes. (**D**) The particle size distribution from SEM images of SPs was estimated using ImageJ's 'Analyze Particles' tool. A binary image is first generated from the SEM image and cleaned up using the drawing tools. Then, an image of the particle outlines is generated, and these are fitted to ellipses using the analysis tool, from which the particle diameters are calculated by approximating the measured ellipse area as the area of a circle.

_s12._ Flow Rate Does Not Change Analyte Accessibility

We tested the effects of flow rate on single-molecule adsorption by carrying out the same overall experimental procedure on untreated Cellulose-B porous particles, flowing 1nM rhodamine 6G solution (in pH 7.33 HEPES) at various pump-driven flow rate settings and collecting 2000 frames at 32 Hz. The flow rates were varied from 0-100 μL/min which are roughly estimated to be at ~700 psi (using Eq. S4, see below), similar to conditions the Reflect columns are operated at, which can be as low as 400 psi, and as high as 6000 psi depending on mobile phase and application (*78*, *79*). We show that changing flow rate does not drastically affect the distribution of analytes within the volume of particles (**fig. S12**A), and in fact can decrease the overall analyte accessibility (**fig. S12**B), which has been previously observed in the literature for ion-exchange columns (*43*). However, we note that the increasing the flow rate (expectedly) linearly increases the number of molecules that are observed over time (**fig. S12**C), since more volume and analytes are passed through during the same given time period. The fact that analytes do not more readily access the inner volume of the porous particles even at purely Brownian motion conditions (0 μL/min; i.e. no pump) suggests that the inaccessibility of the inner volume is not due to our chosen flow rate conditions.

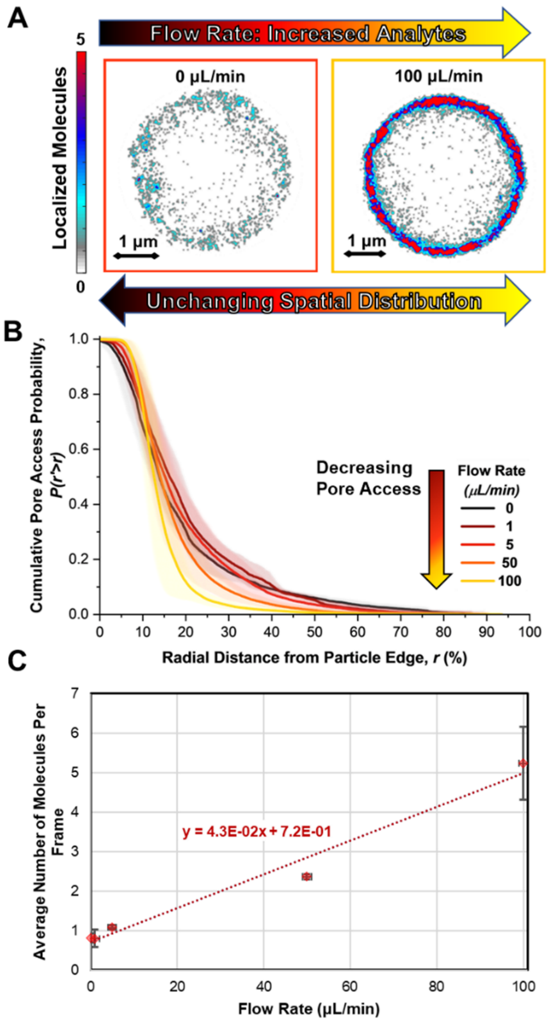

fig. S12. Spatial distribution of analytes within the area of particles remains SPP-like with changing flow rate. (**A**) The super-resolution map of imaged isolated porous Cellulose-B particles shows that more analytes are adsorbed on average, but the spatial distribution is unchanging, with the majority of analytes failing to enter the inner volume of particles. (**B**) The average number of analytes detected increases linearly with flow rate. (**C**) The cumulative distribution of analytes within the particles shows that accessibility to the inner volume decreases with increasing flow rate, but cannot be increased, even under no directional flow. Flow rates 1, 5, 50, and 100 μL/min correspond to approximately 678, 681, 713, 750 psi pressures at the sample.

***s13.* Grace BioLabs Hybriwells Flowcell Pressure Calculation**

To connect our flow-rate based mobile phase conditions inside our flow-cell system (Hybriwell, 13 mm diameter, 0.15 mm depth, Grace Biolabs) to chromatography relevant conditions, we derived a simple method for estimating the pressure inside the cell based on the flow rate set by our syringe pump (NE-1000, New Era Pump Systems Inc.), using 1/32” O.D. x 0.005” I.D. tubing (Tub Peek Red, IDEX Health), and 4.69 mm I.D. syringes (1mL, HSW – Norm Ject, Sigma).

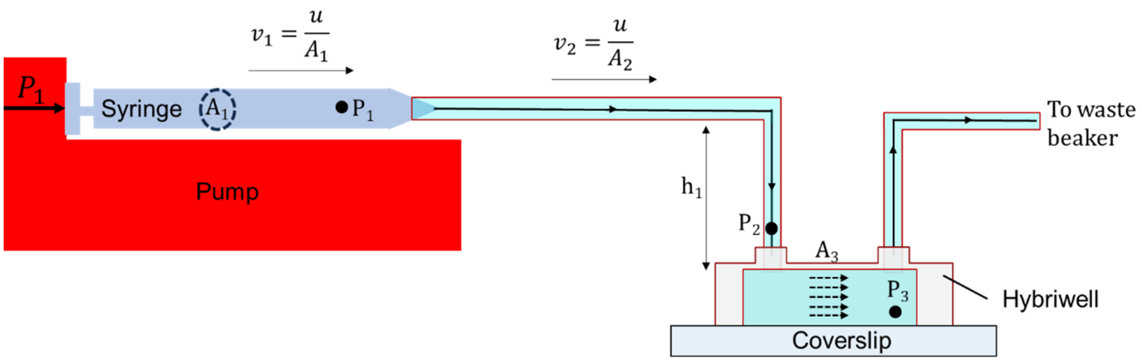

fig. S13. Illustration of the Grace BioLabs Hybriwells setup involving the syringe pump.

We use Bernoulli’s Law to find the pressure in the tubing just before entering the flow cell based on the initial flow conditions from release in the syringe from a height h_1 above the flow cell: $P_1 + \frac{1}{2}\rho v_1^2 + \rho g h_1 = P_2$. Where is the density of solution, and *g* is the gravitational constant. To a first order approximation, we use continuity and the fact that $v = \frac{u}{A_3}$ to write:

$$P_2 = P_1 + \rho g h_1 + \frac{1}{2}\rho u^2\left(\frac{1}{A_1^2} - \frac{1}{A_2^2}\right) \quad \text{Eq. (S2)}$$

Based on the pressure in the tubing just before entering the flow cell, P_2, we calculate the pressure inside the flow cell, P_3 by again applying Bernoulli’s Law. However, we first make the assumption that the fluid motion inside the Hybriwell is laminar and without head loss, and that v_3 inside the flow cell is $v_3 = \frac{u}{A_3}$. This gives:

$$P_3 = P_2 + \rho g h_1 + \frac{1}{2}\rho u^2\left(\frac{1}{A_2^2} - \frac{1}{A_3^2}\right) \quad \text{Eq. (S3)}$$

Substituting Eq. S2 into Eq. S3 finally gives:

$$P_3 = P_1 + \rho g h_1 + \frac{1}{2}\rho u^2\left(\frac{1}{A_1^2} - \frac{1}{A_3^2}\right) \quad \text{Eq. (S4)}$$

P_1 is calculated using data from the syringe pump user manual (NE-1000, New Era Pump Systems Inc), which indicates that the minimum and maximum pressures applied on a 1 mL syringe are 13 psi and 667 psi at flow rates of 0.0121 µL/min and 881 µL/min respectively. We assume a linear relation between the syringe pump pressure and flow rate, from which we can interpolate syringe pump pressures. P_3 is calculated using the parameters in the ’Value’ column of Table S2, estimating the pressure inside the Hybriwells at varying flow rates. Ultimately, the force applied by the syringe primarily determines pressure. Without an applied external force, the pressure in the flow cell is unknown (and motion is predominantly Brownian), but the linear

flow conditions we can achieve are still realistic to what LC systems can operate at (see section *s24*).

Table S2. Variables used in pressure approximation.

Variable	Description	Value
u	Volumetric flow rate of syringe pump	1, 5, 50, 100 µL/min
P_1	Pressure delivered by syringe pump (this is interpolated from data from the manufacturer)	678, 681, 713, 750 psi
A_1	Cross-sectional area of syringe (known from syringe inner diameter)	6.91×10^{-5} m^2
v_1	Flow velocity in syringe	N/A
h_1	Height drop from syringe pump to flow cell	0.30 m
P_2	Pressure in tubing just before entering flow cell	N/A
A_2	Cross-sectional area of tubing (known from tubing inner diameter)	N/A
v_2	Flow velocity in tubing	N/A
P_3	Pressure inside hybriwells	~678, 681, 714, 750 psi
A_3	Cross-sectional area of hybriwells (known from manufacturer details)	1.95×10^{-6} m^2
v_3	Velocity inside hybriwells (we assume laminar flow here)	N/A
ρ	Density of water	1000 kg/m^3
g	Gravitational constant	9.81 m/s^2

***s14.* Refractive Index Matching Does Not Change Visible Analyte Distribution**

We performed super-resolution imaging measurements of rhodamine 6G analyte dissolved in media of matching refractive index to silica and cellulose. A 1 nM rhodamine 6G (99%, Fisher) index-matched solution was prepared by dissolving 60% wt. D-(+)-glucose (>99.5%, Sigma Life Sciences) solid powder in water. Glucose powder was added in small increments to a water solution heated to 50° C, while stirring. The final solution was vortexed and allowed to cool to room temperature before adding solid rhodamine 6G, then the mixture was sonicated and further vortexed to aid dissolution into the viscous solution.

Glucose solutions have been shown to change in refractive index with concentration (*80*), approximately matching the 1.47-1.48 index of cellulose and silica under 532 nm illumination at a ~60+% wt. concentration. Here we only went up to 60% as past that the solution becomes too viscous to effectively flow through our system. Single-molecule imaging was then carried out as in the rest of the text, focusing on untreated Cellulose-B FPPs. The resulting super-resolution maps (**fig. S14**A) show the same spatial distribution as those without index matching media, with little to no analytes reaching the inner volume of the particles. Similarly, the quantified cumulative analyte distributions show no notable change compared to the results done in non-index-matched HEPES buffer solutions (**fig. S14**B). This – along with the fact that analytes are visible and identified within bare silica and Whelk-O1 FPPs – supports that we can reliably image analytes within the volume of these chromatography materials.

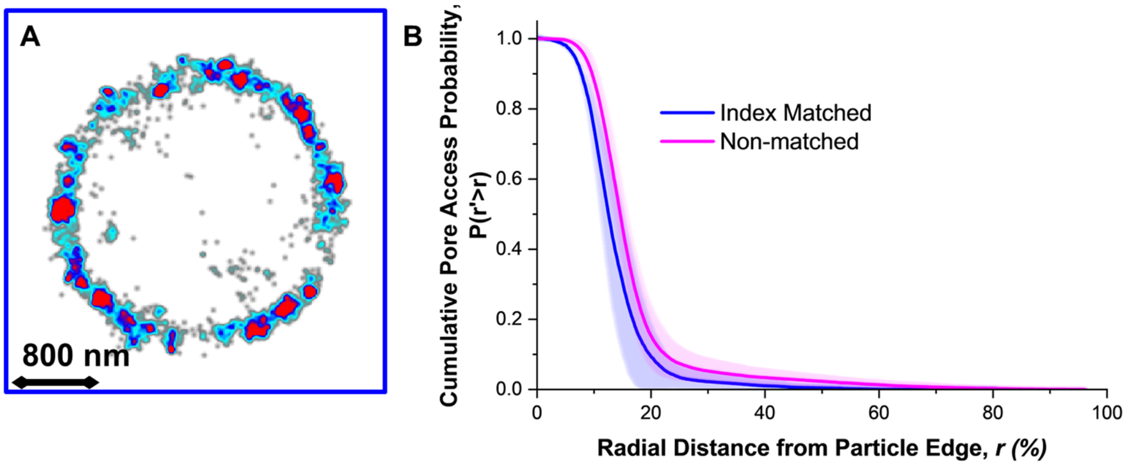

fig. S14. Spatial distribution of analytes within the volume of particles is unchanged with refractive index matching. (**A**) The super-resolution map of imaged isolated porous Cellulose-B particle shows that analytes fail to enter the inner volume of particles. (**B**) The cumulative distribution of analytes under refractive index-matched and non-matched conditions are identical, demonstrating that analytes are not being obscured by the cellulose functionalization.

***s15.* Treatment for Removal of Functionalized Cellulose**
Cellulose-B particles consist of a porous silica (SiO_2) matrix that is coated with cellulose, with functional end groups (*tris(3,5 dimethyl phenyl-carbamate)*). The "fully-porous" stationary phase particles were treated with different concentrations of dimethyl sulfoxide (DMSO; ≥99% pure, MP Biomedicals) dissolved in water. Cellulose-B particles were mixed as 0.1% wt suspensions by vortexing in solutions of 10%, 20%, and 100% v/v DMSO (in H_2O). The mixtures were heated on a hot-plate to ~40˚C, stirring at 250 rpm, and left as such overnight for ~12 hours. Then, the solutions were removed from the hot-plate and centrifuged at 2366 rcf for 10 minutes. The supernatant solution was removed until only the solids at the bottom of the containers (20 mL glass vials in this case) remained. The vials were then refilled with water and vortexed, then stirred at 250 rpm ~5 min. This was followed by another round of centrifuging, removing the supernatant, and another wash (by filling with water and stirring), and repeated at least three times total per solution. Finally, the solutions with treated particles were either used for microscopy by drop-cast liquid sample deposition, or by centrifuging, removing supernatant, and drying at ~40˚C for ~5 hrs to isolate as dry powder for other experiments.

The DMSO solvent treatment successfully removes a some of the functional groups from the surface of Cellulose-B particles. ToF-SIMS images (**fig. S16**) confirm that the bulk of the removal occurs at the outer surface. DMSO by itself is a good solvent for pure cellulose and has been shown to work for cellulose-carbamates. However, the treatment might not be sufficiently harsh to remove all functionalization, and for better cellulose dissolution, DMSO mixtures with other solvents are commonly used (such as DMSO/THF)(*35*). In addition, as our kinetic results indicate (**Fig. 5**), we still maintain selectivity and similar adsorption to the untreated particles. Furthermore, there have been previous studies done using similar cellulose tris (3,5 dimethylphenyl-carbamate) columns for successful enantio-separation in cellulose-dissolving solvents (*81*). As to why the treatment acts more strongly near the entrance, the likely factor is that even the solvent might struggle to penetrate into the particle volume under the untreated conditions. Cellulose-B columns for HPLC measurements followed different DMSO treatment procedure, detailed in section *s20*.

***s16.* 3D Mapping of DMSO (100%) Treated Particle**

3D mapping of Cellulose-B particle after treatment with 100% DMSO solution for 12 hr. Imaging done under the same procedure and conditions as other particles in **Fig. 2** in the main text. 3D slices of the map are included to provide a clearer view of the interior distribution of analytes.

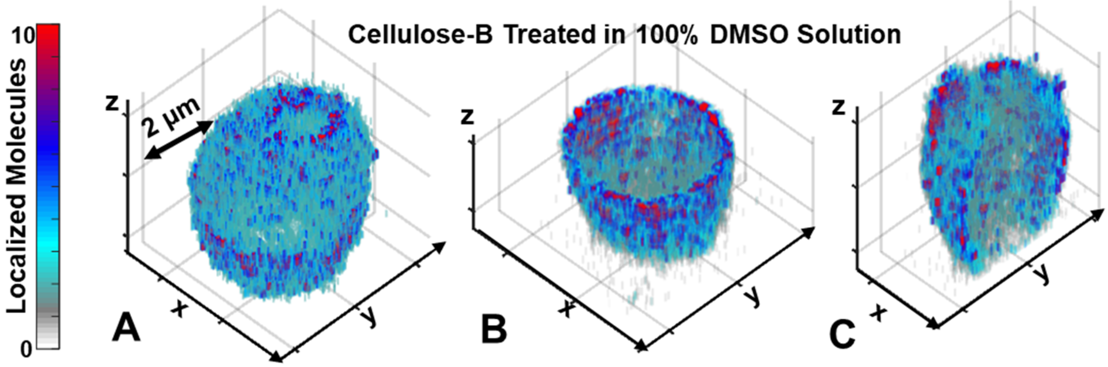

fig. S15. Single-molecule fluorescence characterization of chromatography particles shows regained pore accessibility after treatment with organic solvent. (**A**) The 3D super-resolution map of a rhodamine 6G single molecule adsorptions measured throughout the volume of a Cellulose-B porous chromatography particle. The particle was treated with 100% DMSO solution at 40 ˚C over 12 hrs to remove outer layer of functional groups, regaining accessibility to the inner volume of the particle. (**B**) A horizontal cross section of the particle at the half-height shows visible analytes present within the particle volume. Ring-like features are due to the 250 ± 10 nm spacing between the collected imaging areas (slices). (**C**) Vertical cross section further shows that analyte distribution within the particle volume remains consistent throughout.

***s17.* ToF-SIMS Elemental Mapping Confirms the Presence and Removal of Dense Functionalized Groups in the Inner Volume of the Porous Particles.**

We used the elemental mapping capability of Time of Flight - Secondary Ion Mass Spectrometry (ToF-SIMS) to corroborate our super-resolution microscopy results and further reveal that both the cellulose and chlorine-containing tris(3,5 dimethyl phenyl carbamate) are reduced with DMSO treatment (**fig. S16**). We sought to confirm the spatial distribution of the functional cellulose coating within the chromatography particles, which were imaged after cross sectioning via ion beam milling. Unpacked 5 µm diameter, 1000 Å pore, Cellulose-B (Regis) particles were immobilized as a conductive silver paste (Ted Pella, PELCO Conductive Silver 187). A small drop of conductive silver paint was first deposited on a stainless-steel planar milling blade; the dry sample powder was pressed into it, and then lightly tapped vertically to allow excess to fall off. Ion milling was done on a Gatan Model 693 Ilion+ Precision Cross-Section Ion Milling System, which includes a PIPS Cold Stage Controller. The blade was loaded into the system and pumped down to 3.0 x10^{-5} Torr and allowed to cool (via a liquid nitrogen PIPS cold stage) to -40 °C for 20 min. The sample was then ion milled (UHP Ar background) for 60 min at an accelerating voltage of 5.5 keV. Following milling, the system was vented, and the sample removed.

Elemental mapping (**fig. S16** overlays) was carried out on a Physical Electronics nanoTOF TRIFT V system, with a Primary Liquid Metal Ion Gun (LMIG) set to $^{69}Ga^{+}$, 30 kV, 1 nA (DC). Acquisition was carried out in unbunched negative mode, with e-gun charge compensation, aperture size of 50 µm, and an image scan size of 30 µm. Sample surface was cleaned by light sputtering using a 3 kV, 1000 nA, Ar gas gun, set on a 100 µm x 100 µm area for 15 seconds. Mapping was conducted before and after sputtering to confirm sample stability (immobilization) and cleanliness, with before and after images showing little to no change after sputtering. Ion count signal was collected for ~30 minutes at each sample area, until reaching ~7x10^{6} total ion counts. The secondary electron detector (SED) option on the ToF-SIMS instrument was also used to capture secondary electron images purely as a visual reference of sample morphology (**fig. S16** black and white). Elemental mapping of cross-sectioned particles before (**fig. S16**A) and after DMSO treatment (**fig. S16**B) was done with $^{35}[Cl]^{-}$, $^{12}[C]^{-}$, $^{28}[Si]^{-}$, and $^{16}[O]^{-}$ being our primary reference peaks for monitoring Cl, C, Si, and O ion contents; however, for each measurement, mass range up to 1850 amu was acquired.

ToF-SIMS elemental mapping confirmed the removal of functional groups, and the presence of an outer “shell” that leads to pore blocking in untreated Cellulose-B particles. The accumulation of chlorine-containing groups at the outer surface of particles (**fig. S16**A, overlay) is indicative of the formation of an outer shell of functionalized material that is the likely cause of pore blocking. This shell was then removed by the DMSO solvent treatment (**fig. S16**B, overlay), leaving a more homogenous distribution of functional groups, while allowing analytes and solvent to penetrate into the porous particles. We observe a drop from 75,966 $^{35}[Cl]^{-}$ peak counts (normalized counts: 0.01084) down to 23,725 (normalized counts: 0.00338) after DMSO treatment, indicating a ~70% removal of the visible functional groups.

We finally note that the uneven particle surface deposition and the harsh ion beam milling result in a large difference between the detectable distribution of Cl counts, given different depth of sectioning, and distances from the detector. This is further complicated by the small amount of chlorine groups present in the sample to begin with, which are difficult to map out without long

collection times. Regardless, we were able to observe a clear decrease in Cl groups after DMSO treatment, as well as a shift in the overall distribution, where the high density of functional groups near the outer edge of the particles was removed.

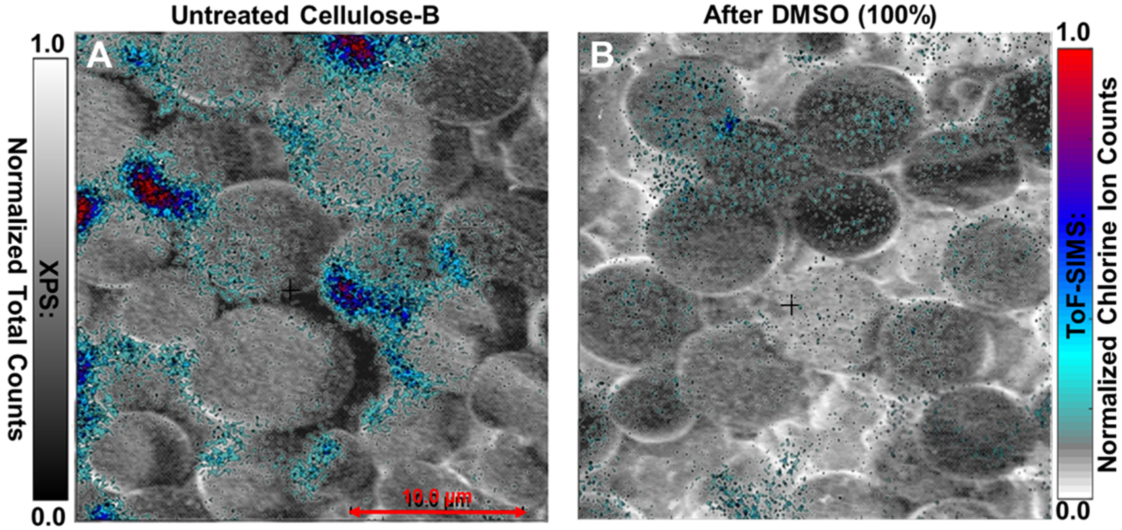

fig. S16. ToF-SIMS elemental mapping of chromatography particle cross-sections before and after treatment with DMSO solvent shows removal of an outer functionalized shell, and reduced degree of functionalization. (**A**) The accumulation of chlorine-containing groups at the outer surface of particles (overlay) is indicative of the formation of an outer shell of functionalized material that is the likely cause of pore blocking. (**B**) This shell is removed by the DMSO solvent treatment (overlay), leaving less dense, but more homogenous distribution of functional groups. Chromatography particles were cross sectioned by immobilizing on conductive silver paste then ion milling for 60 minutes at an accelerating voltage of 5.5 keV

s18. Free Energy Derived from Adsorption Kinetics

The free energy of adsorption can be experimentally estimated from the cumulative dwell and association time distribution of single-molecule analyte observations. The measured dwell time distribution for sorption sites is fitted to a single-term decaying exponential for weakly adsorbing sites, and a two-term decaying exponential for strong specific adsorption (*82*).

$$P(t' > t) = C_1 e^{-t \cdot k_1} + C_2 e^{-t \cdot k_2} \qquad \text{Eq. (S5)}$$

The rate constants (k_1, k_2) of the exponentials correspond to desorption rates for weak and strong specific adsorption, respectively. The same can then be applied to the association time distribution data, giving rates for adsorption. These can then be converted to free energy (ΔG) terms by the equation:

$$\Delta G = -RTln(k_{eq}) \qquad \text{Eq. (S6)}$$

where R is the gas constant, and T is the temperature (in Kelvin) (*39*) and k_{eq} is the ratio of the adsorption (k_a) over the desorption (k_d) rate constant, obtained from the association and dwell time distributions, respectively. Here we took T to be 20 °C (293K). Following this procedure, we get a minimum measured free energy of -2.9 ± 0.9 kJ/mol for specific adsorption, and -11.4 ± 0.6 kJ/mol maximum for non-specific adsorption, consistent with hydrogen bonding, hydrophobic, or electrostatic interactions between the analyte and stationary phase.

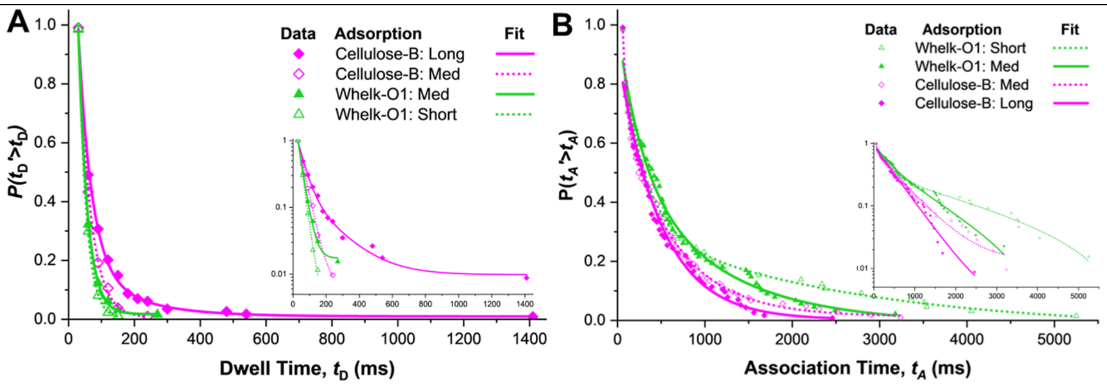

C

Site	Adsorption	k_d (1/ms)	σ_d (1/ms)	k_a (1/ms)	σ_a (1/ms)	ΔG (kJ/mol)	σ_G (kJ/mol)
Cellulose-B	***Long**: Specific*	6.2E-03	2.2E-03	1.9E-03	8.5E-05	-2.9	0.9
	***Med**: Non-specific*	2.7E-02	1.0E-03	1.7E-03	4.6E-05	-6.8	0.1
Whelk-O1	***Med**: Non-specific*	3.6E-02	1.3E-03	9.3E-04	3.1E-04	-8.9	0.8
	***Short**: Non-specific*	4.2E-02	1.5E-03	3.9E-04	8.8E-05	-11.4	0.6

fig. S17. Free energy derived from single-molecule measurements of adsorption kinetics. (**A**) Experimental kinetic adsorption data (measured as dwell times) are plotted as cumulative distributions. These can be modeled as and fit to two-term decaying exponentials, corresponding to weak and strong specific adsorption, respectively. (**B**) Association time distributions are simultaneously obtained from the measured time between adsorptions. Insets show semi-log versions of the plots. (**B**) The results of the fitted exponentials can then be converted to free energy (*ΔG*) by using the adsorption and desorption rates constants obtained from the fits of the time distributions.

***s19.* Lévy Process Model Representation of Elution**

Measured single-molecule adsorption kinetics in porous stationary phase particles were used to model and reveal differences in larger scale chromatographic elution**.** Elution through a chromatography column is modeled by as a stochastic process based on Lévy stochastic processes description. This approach relies on a “real discrete distribution” of sorption times obtained by single-molecule dynamics observation and is well described in Pasti et. al (*41*), with example application in Kisley et. al (*83*).

Briefly, the stochastic model of elution starts by converting the cumulative (Poisson) distribution of the duration of adsorption/desorption events to the frequency domain (ω), then accounting for the discontinuities by using the Lévy representation. The distribution (*f*) of time spent in the stationary phase (dwell time, t_D) is related to characteristic function (ϕ) formalism as:

$$\phi(t_D, \omega, t_A) = \exp\,[r_m \sum_{i=1}^{i=k} (e^{i\omega t_{D,i}} - 1) \cdot f(t_{D,i})] \qquad \text{Eq. (S7)}$$

where t_A is the time spent in the mobile phase (i.e. association/desorption time), for an analyte that has adsorbed to the stationary phase r_m times, and *k* is the index of discrete set of desorption times. By performing a Fourier transform, Eq. S7 is converted to the time domain, allowing for simulation of the chromatographic elution peak. Dwell times of single molecule adsorption are generated by collecting 2000+ frame movies of dye molecules interacting with the porous materials at set vertical positions at the approximate half-height of the particles. The asymmetry of the elution peaks generated from the Lévy process representation of chromatography adsorption developed by Pasti et al.(*41*) is dependent on the chosen value r_m. However, this r_m value is difficult to equate to the measured single-molecule adsorption events that occur in an imaged area, so it is often chosen empirically. Here we observed that this asymmetry decays exponentially with increasing r_m, down to a floor of ~1.1 (**fig. S18**). The minimum asymmetry is approximately reached when r_m is of comparable magnitude to the number observed time steps used in the model. To reduce variability on our model elution curves we selected an r_m value of 1000, at which point the decay curve for asymmetry begins to plateau.

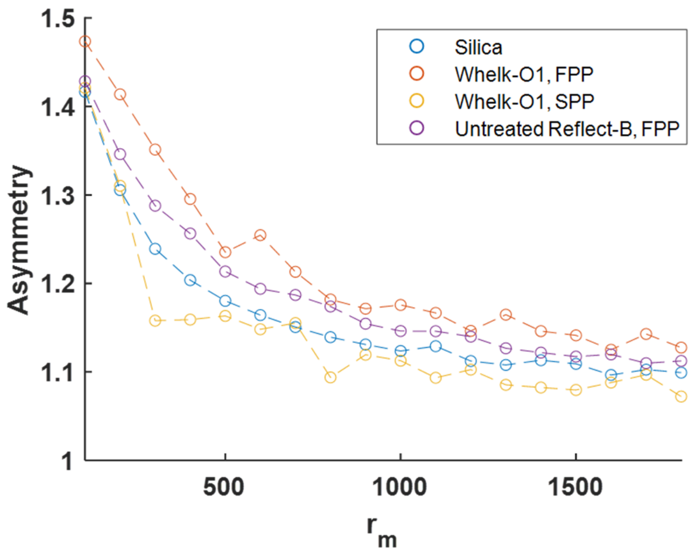

fig. S18. Levy process model chromatographic peaks decrease in asymmetry with increasing average number of adsorption events. The asymmetry of the elution peaks generated from the Levy process model representation of chromatography adsorption developed by Pasti et al.(*41*) is dependent on the chosen value r_m, which corresponds to the average number of a given molecule adsorbs over the elution through a column. We observe that this asymmetry decays exponentially with increasing r_m, down to a floor of 1.1. The minimum asymmetry is approximately reached when r_m is of comparable magnitude to the number observed time steps used in the model, which were 3000 (frames) in this case.

***s20.* HPLC: Bulk Chromatography HPLC Measurements and Comparison to Single Molecule Results**

All bulk chromatography experiments were performed on a Shimadzu liquid chromatography instrument (LC-20AD Prominence) with photodiode array detection (SPD-M20A, Prominence) with an isocratic mobile phase of 70% ethanol (EtOH, 200 proof, ACS grade from Pharmaco) and 30% HEPES (20 mM, pH 7.33, 2-[4-(2-hydroxyethyl)-1-piperazinyl]-ethane sulfonic acid, high purity from VWR).

A REFLECT C-Cellulose B (5 µm, 5 cm length x 4.6 mm diameter from Regis Technologies) column was pre-treated with dimethyl sulfoxide (DMSO, HPLC grade from Fisher) at 30 ºC as follows. First, 20 vol% DMSO:H2O was flowed through the column at 0.1 mL/min for 10 min, 0.2 mL/min for 10 min, 0.3 mL/min for 3 hrs, and 0.4 mL/min for 10 min; the 20 vol% DMSO:H2O was then left in the column, stagnant, for 18 hours. This slow increase of flow rate was found necessary due to excessive column backpressure if high (>0.3 mL/min) flow rates were initially employed. Finally, the column was flushed with 20 vol% DMSO:H2O at 0.3 mL/min for 30 min. Prior to experiments, the DMSO-treated column was rinsed with mobile phase (70% EtOH:30% HEPES) at 0.1 mL/min for 2 hrs then at 0.3 mL/min for 2 hrs. The control (untreated) column was rinsed with mobile phase (70% EtOH: 30% HEPES) at 0.3 mL/min for at least 2 hrs prior to experiments.

Rhodamine 6G (3 µL, 100 µM in HEPES buffer, from Biotang Inc) was run on each column (n=4 replicate injections) with an isocratic mobile phase of 70% EtOH: 30% HEPES, a flow rate of 0.5 mL/min, and a column temperature of 30 ºC. Detection was accomplished using a photodiode array over the range 200 – 600 nm at 5 Hz. The resulting chromatograms were visualized in Shimadzu Lab Solutions software, extracted at 530 nm (4 nm bandwidth), exported, and further analyzed with Origin 2022.

Comparison to the model elution curves generated from single-molecule measurements show remarkably similar peak shapes, with comparable tailing (**fig. S19**). Both the model and HPLC measured elutions show asymmetries (measured as the ratio of the width of the right side of the peak over the width of the left side), favoring the right side of the curve (known as tailing), which are associated with strong retention of analyte in the stationary phase. Both also show little to no change in the asymmetry of the curves before and after treatment of Cellulose-B particles with DMSO, supporting our conclusions that the particles remain sufficiently functionalized to achieve a separation even after treatment. However, the degree of tailing differs between the model and HPLC measurements, where we find that the experimental bulk asymmetry (up to a degree of 2) can be up to 1.5x what the model predicts (up to a degree of 1.3). Similarly, the change in broadening is lower for the model data. Where the model shows an 11% decrease in peak width, the HPLC results show broadening with an increase of 50% peak width. We can attribute these differences to the limitation of the single-molecule model, where it only accounts for adsorption mass transfer behavior, while the elution peak in a column is influenced by many other factors such as convection, Eddy, and longitudinal diffusion due to column length and packing. Furthermore, as mentioned in the main text, differences in the DMSO treatment in column vs. for unpacked materials can yield different degrees of de-functionalization.

In typical chromatographic metrics, we can apply the full-width-half-max (FWHM) equation (Eq. S8) for theoretical plate height, *HETP*, due to the asymmetry of the peaks:

$$HETP = L \cdot \frac{(t_r - t_u)^2}{5.54 \cdot (w_{0.5})} = L/N \quad \text{Eq. (S8)}$$

Where $w_{0.5}$ is the FWHM, t_r is the analyte elution time, t_u is the void elution time (only applies for HPLC experiment), N is the number of plates, and L is the column length (50 mm). This yields the *HETP* before and after DMSO treatment of 0.33 to 0.29 for single-molecule kinetics and 0.39 to 2.79 for HPLC, respectively. As mentioned, due to limitations of accounting for increasing intra-particle diffusion and changes to other modes of diffusion, these model peaks do not currently reflect broadening trends in HPLC, but instead can inform elution speed and asymmetry (due to rare, strong adsorptions). Regardless, these results overall indicate that solvent treatment 1) reduces the degree of functionalization and 2) causes peak broadening, while 3) simultaneously speeding up elution. Balancing these three variables could be the key to optimizing separation. We suspect that despite the increased available surface area, the DMSO treatment sufficiently reduced the density of functionalized sites to cause an overall loss in retention. Similarly, the increased available intra-particle volume results in longer diffusion trajectories, causing further peak broadening. Overall, this means that product recovery will be affected by both shorter processing times, but reduced resolution, which is a difficult combination of variables to optimize. In this case, the treatment likely reduces product recovery, but speeds up elution.

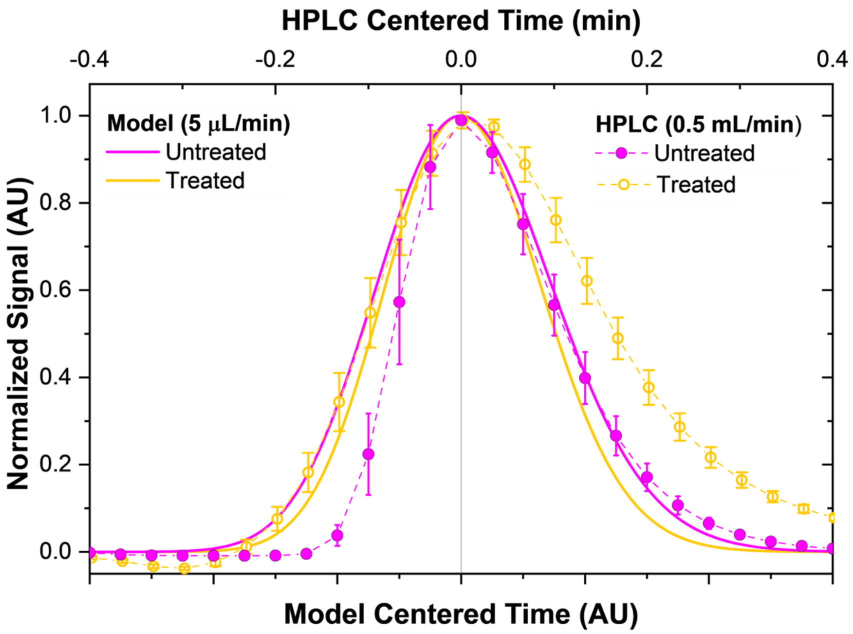

fig. S19. HPLC system elution measurements with packed Cellulose-B columns before and after treatment with DMSO solvent show similar peak shape and tailing as those generated with our single-molecule model of elution curves.

***s21.* Testing the Effects of pH on Accessibility.**

We tested the effect of different pH conditions on pore accessibility of the Cellulose-B stationary phases. We prepared 20 mM acetate buffers by mixing sodium acetate (trihydrate solid salt, Fisher) and acetic acid (liquid, glacial, Fisher) in Type I water, changing pH by changing the ratio of salts, then titrating with 20 mM HCl or NaOH as needed to fine tune the desired pH. The titrating solutions were prepared by diluting 1M HCl (Fisher) and 1M NaOH (Fisher). Similarly, bicarbonate-carbonate buffers were prepared by mixing sodium bicarbonate (solid powder, Fisher) and sodium carbonate (solid powder, anhydrous, Fisher) in water, then titrating with 20 mM HCl and NaOH. The resulting buffers used were measured as pH 3.67 and 5.71 acetate buffer, and pH 9.20 and 11.04 carbonate-bicarbonate buffers. Then, 100 nM rhodamine 6G (99%, Fisher) dissolved in water was mixed in with the buffers to create 1 nM rhodamine 6G buffer solutions. Samples of untreated Cellulose-B functionalized particles were prepared as explained in section *s2*, and imaging was done as explained in section *s3*, but using the varied pH buffers instead. As shown in **fig. S20**A, we found little to no change in the spatial distribution of analytes under the varying pH conditions, with no sign of positive improvement to dye accessibility in any tested conditions (**fig. S20**B), matching the observed distribution when using pH 7.33, 20 mM HEPES buffer.

Importantly, in these tests we cover a range of relevant conditions for the use of Cellulose-B particles. Cellulose tris 3,5 (dimethylphenycarbamate) chromatography columns show best performance in pH 3.5-6.5 (*24*, *78*), but are viable up to pH ~9 (*84*). As such, here we tested both favorable and unfavorable pH conditions. Furthermore, the recommended operating conditions are typically at low salt concentrations (<0.2 M) for most separations (*24*), showing decreasing separation performance with increasing salt content. Here we are working at lower 0.02 M buffer concentrations, reducing the effects of surface protonation, allowing for better adsorption of our cationic rhodamine 6G dye since it does not need to compete for adsorption sites with other charged species in solution (*24*).

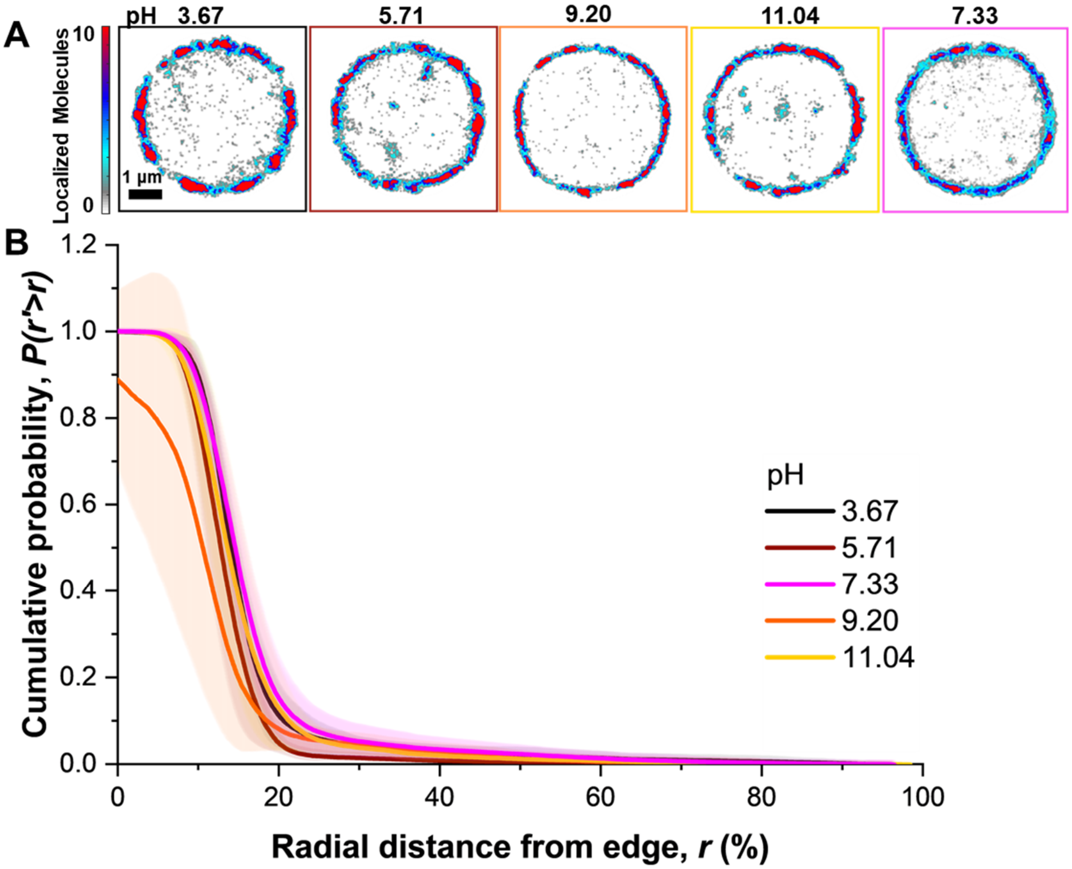

fig. S20. Changing pH conditions does not favorably change the distribution of analytes within the porous functionalized particles.

(**A**) As solvent pH conditions increase from 3.67 up to 11.04, analyte affinity slightly varies, (**B**) but the overall distribution throughout the volume of particles does not drastically change.

***s22.* Testing the Effect of Dye Charge.**

We tested the effect of analyte charge by imaging the distribution of the anionic dye fluorescein in Cellulose-B particles, using the same varying pH buffers presented in section *s21*. Fluorescein powder (Fluka Analytical; Honeywell) was first dissolved in pure DMSO as a 10 mM solution, then diluted in water to a 1.0 μM dye solution stock. After, the dye was further diluted to 100, 10, and 1 nM solutions in pH 3.67 and 5.71 acetate buffer, and to pH 9.20 and 11.04 in carbonate-bicarbonate buffers. As exemplified in **fig. S21**, we found that under none of the tested conditions would the dye favorably adsorb to the surface (**fig. S21**A). Short-lived and rare occurrences of dye adsorption could be found and observed, but not sufficiently to obtain good statistics of single-molecule events (**fig. S21**B), and not-sufficiently to generate detailed super-resolution maps of the adsorption. Increasing dye concentrations in solution from 1 nM up to 100 nM only led to increasing background signal without more molecules visibly adsorbing to the stationary phase (**fig. S21**C). This suggests strong repulsion of the negatively charged fluorescein dye by the stationary phase, and further supports the assumed electrostatic attraction of the positively charged rhodamine 6G to the negatively charged groups of the silica surface and Cellulose-B.

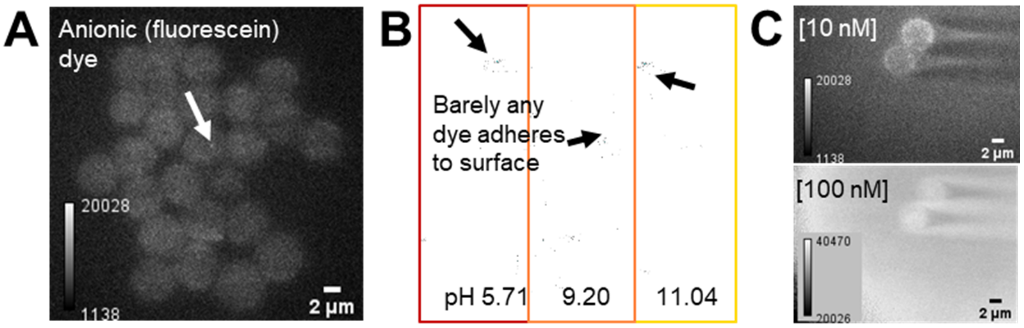

fig. S21. Anionic dye does not favorably adsorb unto Cellulose-B functionalized particles**.** The partially negatively charged fluorescein is largely repelled from the functionalized chromatography particles. (**A**) The anionic fluorescent dye can rarely be observed adhering to the material. (**B**) Single-molecule localization analysis shows that some dye molecules adhere over time at varying pH conditions, but not sufficiently to draw good statistics. (**C**) Increasing dye concentrations in solution only lead to increased background fluorescence signal away from the stationary phases, without improving the amount of observed dye adsorption events.

---

s23. **Grouped Particle Pore Accessibility Behavior.**
Throughout all presented experiments, grouped stationary phase particles were also observed deposited on the surface of the microscopy slides within the flow cell. These particles would naturally aggregate and sometimes further pack together under flow conditions. To further support our pore accessibility observations, we exemplify in **fig. S22** the pore blocking behavior that is also observed when imaging grouped (i.e. packed) untreated Cellulose-B functionalized particles. Similarly to what has been shown throughout the rest of the text, such pore blocking behavior also limits inter-particle diffusion, reducing the number of adsorptions deeper into the particle groupings. Meanwhile, pore-blocking is not present for non-functionalized (i.e. bare silica) and DMSO treated (i.e. partially de-functionalized) Cellulose-B particles, where intra-particle mass transfer occurs more readily. This is consistent across all measurements, and overall, we have made observations of 1,000’s of particles and 1,000,000’s of single molecule events. The lack of pore blocking when particles are grouped supports that sufficient intra-particle solvation occurs in non-functionalized and DMSO treated particles, and that flow conditions are not the primary cause for the observed surface-limited adsorption seen on functionalized materials.

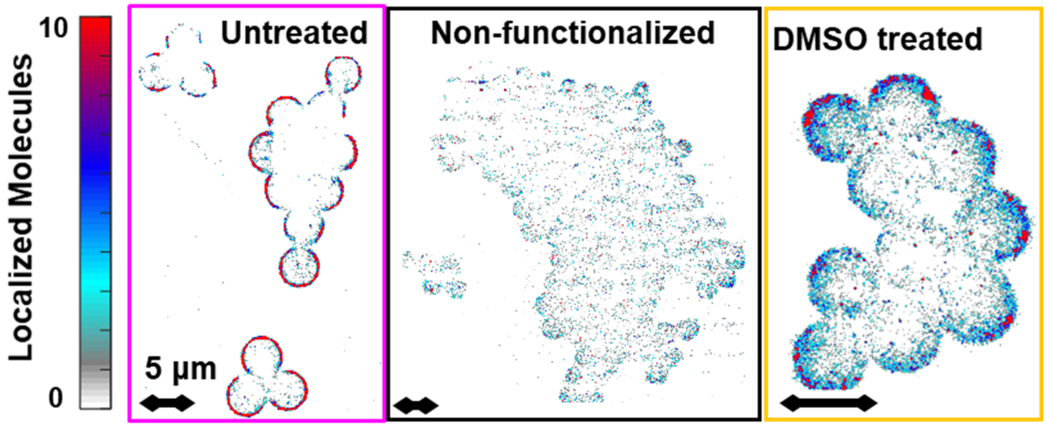

fig. S22. Pore accessibility trends observed in isolated single stationary phases remain with grouped/packed particles. Low pore accessibility is still observed in grouped Cellulose-B functionalized stationary phase particles, reducing intra-particle diffusion. Bare silica non-functionalized, or DMSO treated particles do not show the same pore blocking behavior, even when grouped, and intra-particle diffusion readily occurs.

***s24.* Estimating Linear Flow Velocities.**
Here we estimate the linear flow velocities within our chromatography column and our flow-cell, to connect our microscopy solvent flow rate conditions to those used in HPLC. As stated in section *s2*, our Hybriwell flow cell dimensions are 13 mm diameter, 0.15 mm depth (*h*), and we use primarily a 5 µL/min operating flow rate. The flow-cell has two outlet and inlet apertures that are ~10 mm in diameter, set 8 mm (inner-to-inner circumference) apart. It is within this separation that we image the deposited stationary phases. The deposition can be somewhat random due to relying on polar interactions for immobilization, but we can estimate the cross-sectional area as a range of values defined by slices of cylindrical well. For a radius (*r*) of 7.5 mm, and a limited range of deposition (*x*) defined from the center of the circumference, we can define the cross-sectional radial length (*l*) as:

$$l = \sqrt{r^2 - x^2}\,; r = 0.65\ cm, x = 0\ to\ 0.4\ cm \qquad \text{Eq. (S9)}$$

From which we define the flow cell cross sectional area, A_X:

$$A_x = h \cdot 2l \qquad \text{Eq. (S10)}$$

Given that the amount of deposited stationary phase material is very low, we directly estimate that for a given applied flow rate f_r (assuming 1 mL/min = cm^3/min, for solvent density ~1), the linear velocity in the Hybriwell cross section (v_x) is defined as:

$$v_x = \frac{f_r}{A_x} \qquad \text{Eq. (S11)}$$

This gives us a range of cross-sectional areas 0.0154 to 0.0195 cm^2, and therefore linear flow velocities of 0.26 to 0.33 cm/min for an applied flow rate of 5µL/min.

Similarly, we can estimate the linear flow velocity within our HPLC columns that have dimension of 5.0 cm length, and 4.6 mm inner diameter. For the cylindrical shape of the columns, this gives a cross-sectional area (A_c) of ~ 0.17 cm^2. Assuming a ~0.6 occupied volume (V_p) filled with stationary phase material, we can estimate the linear velocity in the columns (v_c) for a given flow rate (f_r) as:

$$v_c = \frac{f_r}{(A_c \cdot V_p)} \qquad \text{Eq. (S12)}$$

This gives us an estimated linear velocity of ~ 5.0 cm/min for an applied flow rate of 0.5 mL/min on the column. This means that the linear velocity experienced by solvent within the Hybriwell (~0.26 cm/min at 5 µL/min) is actually lower than in the column for the examples demonstrated in **Fig. 5** and **fig. S19**, by a factor of ~20x. However, as we demonstrate in section *s12*, we can achieve the same linear velocities between the two systems at higher (namely ~5.0 cm/min at 100 µL/min) applied flow rates in the Hybriwell, but, as shown in **fig. S12**, and hinted at in literature (*43*), increasing flow rates results in decreasing pore access.

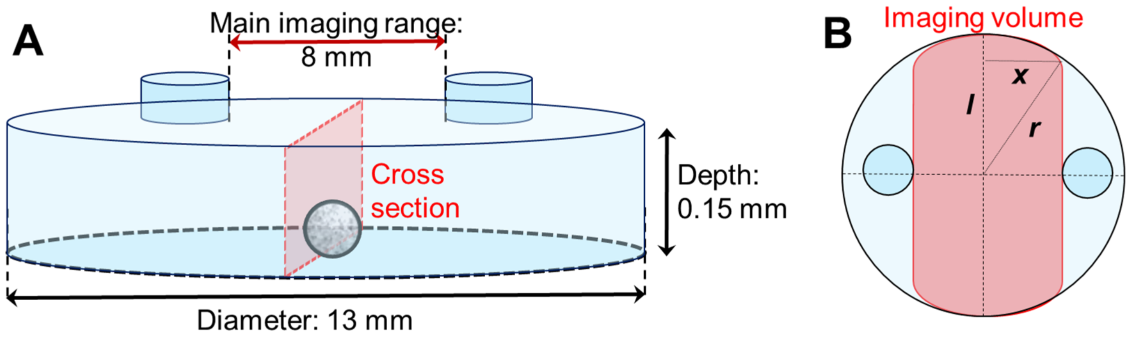

fig. S23. Linear flow velocity can be estimated based on the cross-sectional area where deposited stationary phases are imaged.

***s25.* Available Data and Caption for Data S1**

Data S1. All data used to generate the Main Text figures (Figs.1-5) and associated results. Data are provided in the supplementary materials spreadsheet document Data S1. Within the spreadsheet document we provide:

- Fig 2B: The intensity map data for the Cellulose-B particle 2D cross-section.
- Fig 2C: The intensity map data for the Silica particle 2D cross-section.
- Fig 2D: The intensity map data for the Whelk-O1 SPP particle 2D cross-section.
- Fig 3C: The molecule radial cumulative distribution (*P(r'>r)*, *r* is radial % distance) data for each individual particle (P1, P2, P3) and the averages presented in **Fig. 3C**.
- fig S8A: The nitrogen adsorption data plotted in **fig. S8A**.
- Fig. 3D & fig. S8B: The accessible area and BET surface area values that were plotted as bar-plots in **Fig. 3D**, and **fig. S8B**.
- Fig 4B: The molecule radial cumulative distribution (*P(r'>r)*, *r* is radial % distance) data for each individual particle (P1, P2, P3) and the averages presented in **Fig. 4B**. The Cellulose-B and Silica distributions are the same as **Fig. 3C**.
- Fig 4C: The accessible areas that were plotted as a bar-plot in **Fig. 4C**.
- Fig 5A: The molecule dwell time cumulative distribution (*P(t'>)*, *t* is dwell time in ms) data for **Fig. 5A**.
- Fig 5B: The model elution curve data presented in **Fig 5B**, and the expeimental HPLC elution data and standard deviation (fom 3 runs) obtained and plotted in **Fig 5B**.

In addition, raw .tiff microscopy movies are provided (labeled in accordance to figure numbers) at Zenodo DOI: 10.5281/zenodo.13697196.

REFERENCES AND NOTES

1. National Academies of Sciences, Engineering, and Medicine, Division on Earth and Life Studies, Board on Chemical Sciences and Technology, Committee on a Research Agenda for a New Era in Separation Science, *A Research Agenda for Transforming Separation Science* (National Academies Press, 2019).

2. D. S. Sholl, R. P. Lively, Seven chemical separations to change the world. *Nature* **532**, 435–437 (2016).

3. J. C. Giddings, Dynamics of Zone Spreading, in *Dynamics of Chromatography: Principles and Theory* (Marcel Dekker Inc., ed. 1, 1965), pp. 13–94.

4. K. Sing, The use of nitrogen adsorption for the characterisation of porous materials. *Colloids Surf. A Physicochem. Eng. Asp.* **187-188**, 3–9 (2001).

5. S. Fu, Q. Fang, A. Li, Z. Li, J. Han, X. Dang, W. Han, Accurate characterization of full pore size distribution of tight sandstones by low-temperature nitrogen gas adsorption and high-pressure mercury intrusion combination method. *Energy Sci. Eng.* **9**, 80–100 (2021).

6. B. M. Wagner, S. A. Schuster, B. E. Boyes, T. J. Shields, W. L. Miles, M. J. Haynes, R. E. Moran, J. J. Kirkland, M. R. Schure, Superficially porous particles with 1000 Å pores for large biomolecule high performance liquid chromatography and polymer size exclusion chromatography. *J. Chromatogr. A* **1489**, 75–85 (2017).

7. Y. Min, B. Jiang, C. Wu, S. Xia, X. Zhang, Z. Liang, L. Zhang, Y. Zhang, 1.9 μm Superficially porous packing material with radially oriented pores and tailored pore size for ultra-fast separation of small molecules and biomolecules. *J. Chromatogr. A* **1356**, 148–156 (2014).

8. L. Hagel, M. Östberg, T. Andersson, Apparent pore size distributions of chromatography media. *J. Chromatogr. A* **743**, 33–42 (1996).

9. G. Carta, A. Jungbauer, *Protein Chromatography: Process Development and Scale-Up* (Wiley-VCH, ed. 2, 2020).

10. S. Misra, M. F. Wahab, D. C. Patel, D. W. Armstrong, The utility of statistical moments in chromatography using trapezoidal and Simpson's rules of peak integration. *J. Sep. Sci.* **42**, 1644–1657 (2019).

11. K. Miyabe, Moment theory for kinetic study of chromatography. *TrAC Trends Anal. Chem.* **81**, 79–86 (2016).

12. K. Miyabe, A. Ishitobi, K. Hiyama, F. Kubotani, Moment analysis method for measurement of reaction equilibrium and rate constants by using high-performance liquid chromatography. *Anal. Chem.* **96**, 4553–4561 (2024).

13. S. Qamar, A. Seidel-Morgenstern, Extending the potential of moment analysis in chromatography. *TrAC Trends Anal. Chem.* **81**, 87–101 (2016).

14. K. Miyabe, A. Arai, M. Ishizuka, Moment theory of chromatography for the analysis of reaction kinetics of intermolecular interactions. *Anal. Chem.* **93**, 10365–10371 (2021).

15. L. D. C. Bishop, A. Misiura, N. A. Moringo, C. F. Landes, Unraveling peak asymmetry in chromatography through stochastic theory powered Monte Carlo simulations. *J. Chromatogr. A* **1625**, 461323 (2020).

16. A. Felinger, Molecular dynamic theories in chromatography. *J. Chromatogr. A* **1184**, 20–41 (2008).

17. J. Urban, Pore volume accessibility of particulate and monolithic stationary phases. *J. Chromatogr. A* **1396**, 54–61 (2015).

18. K. L. Nguyen, V. Wernert, A. M. Lopes, L. Sorbier, R. Denoyel, Effect of tortuosity on diffusion of polystyrenes through chromatographic columns filled with fully porous and porous – shell particles and monoliths. *Microporous Mesoporous Mater.* **293**, 109776 (2020).

19. R. Monge Neria, L. Kisley, Single-molecule imaging in commercial stationary phase particles using highly inclined and laminated optical sheet microscopy. *Anal. Chem.* **95**, 2245–2252 (2023).

20. M. J. Wirth, D. J. Swinton, M. D. Ludes, Adsorption and diffusion of single molecules at chromatographic interfaces. *J. Phys. Chem. B* **107**, 6258–6268 (2003).

21. J. T. Cooper, E. M. Peterson, J. M. Harris, Fluorescence imaging of single-molecule retention trajectories in reversed-phase chromatographic particles. *Anal. Chem.* **85**, 9363–9370 (2013).

22. F. C. Hendriks, F. Meirer, A. V. Kubarev, Z. Ristanović, M. B. J. Roeffaers, E. T. C. Vogt, P. C. A. Bruijnincx, B. M. Weckhuysen, Single-molecule fluorescence microscopy reveals local diffusion coefficients in the pore network of an individual catalyst particle. *J. Am. Chem. Soc.* **139**, 13632–13635 (2017).

23. W. Calabrase, L. D. C. Bishop, C. Dutta, A. Misiura, C. F. Landes, L. Kisley, Transforming separation science with ingle-molecule methods. *Anal. Chem.* **92**, 13622–13629 (2020).

24. J. G. Hou, Y. L. Wang, C. X. Li, X. Q. Han, J. Z. Gao, J. W. Kang, Reversed-phase separations on cellulose tris(3,5- dimethylphenylcarbamate) chiral stationary phase. *Chromatographia* **50**, 89–95 (1999).

25. Q. Wei, H. Su, D. Gao, S. Wang, HPLC with cellulose tris (3,5-dimethylphenylcarbamate) chiral stationary phase: Influence of coating times and coating amount on chiral discrimination. *Chirality* **31**, 164–173 (2019).

26. A. J. Bain, P. Chandna, G. Butcher, J. Bryant, Picosecond polarized fluorescence studies of anisotropic fluid media. II. Experimental studies of molecular order and motion in jet aligned rhodamine 6G and resorufin solutions. *J. Chem. Phys.* **112**, 10435–10449 (2000).

27. C. B. Müller, A. Loman, V. Pacheco, F. Koberling, D. Willbold, W. Richtering, J. Enderlein, Precise measurement of diffusion by multi-color dual-focus fluorescence correlation spectroscopy. *Europhys. Lett.* **83**, 46001 (2008).

28. Q. Tian, C. Lv, P. Wang, L. Ren, J. Qiu, L. Li, Z. Zhou, Enantiomeric separation of chiral pesticides by high performance liquid chromatography on cellulose tris-3,5-dimethyl carbamate stationary phase under reversed phase conditions. *J. Sep. Sci.* **30**, 310–321 (2007).

29. S. Khater, Y. Zhang, C. West, In-depth characterization of six cellulose tris-(3,5-dimethylphenylcarbamate) chiral stationary phases in supercritical fluid chromatography. *J. Chromatogr. A* **1303**, 83–93 (2013).

30. D. M. Schlipf, S. E. Rankin, B. L. Knutson, Selective external surface functionalization of large-pore silica materials capable of protein loading. *Microporous Mesoporous Mater.* **244**, 199–207 (2017).

31. A. Katiyar, N. G. Pinto, Visualization of size-selective protein separations on spherical mesoporous silicates. *Small* **2**, 644–648 (2006).

32. H. C. Daniel, C. A. Lucy, "High-performance liquid chromatography, Figure 25-5" in *Quantitative chemical analysis* (W. H. Freeman & Company, ed. 9, 2015), p. 671.

33. S. Cao, Q. Ma, Y. Liu, J. Zhang, Z. Wang, Cellulose tris-(3,5-dimethyl phenyl carbamate) as a chiral stationary phase for enantiomeric determination of ofloxacin enantiomers and molecular docking study on the chiral separation mechanism. *New J. Chem.* **46**, 9704–9709 (2022).

34. G. K. E. Scriba, Chiral recognition mechanisms in analytical separation sciences. *Chromatographia* **75**, 815–838 (2012).

35. T. Liebert, Cellulose solvents-remarkable history, bright future. *ACS Symp. Ser.* **1033**, 3–54 (2010).

36. J. C. Giddings, H. Eyring, A molecular dynamic theory of chromatography. *J. Phys. Chem.* **59**, 416–421 (1955).

37. L. Kisley, J. Chen, A. P. Mansur, B. Shuang, K. Kourentzi, M.-V. Poongavanam, W.-H. Chen, S. Dhamane, R. C. Willson, C. F. Landes, Unified superresolution experiments and stochastic theory provide mechanistic insight into protein ion-exchange adsorptive separations. *Proc. Natl. Acad. Sci. U.S.A.* **111**, 2075–2080 (2014).

38. R. Milo, R. Phillips, in *Cell Biology by the Numbers* (Garland Science, ed. 1, 2015), p. 400.

39. C. Zhan, G. Wang, X.-G. Zhang, Z.-H. Li, J.-Y. Wei, Y. Si, Y. Yang, W. Hong, Z.-Q. Tian, Single-molecule measurement of adsorption free energy at the solid–liquid interface. *Angew. Chem. Int. Ed. Engl.* **58**, 14534–14538 (2019).

40. C. Dutta, A. Misiura, L. D. C. Bishop, A. B. Marciel, L. Kisley, C. F. Landes, Toward protein chromatography by design: Stochastic theory, single-molecule parameter control, and stimuli-responsive materials. *J. Phys. Chem. C* **126**, 18587–18595 (2022).

41. L. Pasti, A. Cavazzini, A. Felinger, M. Martin, F. Dondi, Single-molecule observation and chromatography unified by Lévy process representation. *Anal. Chem.* **77**, 2524–2535 (2005).

42. M. Kruk, B. Dufour, E. B. Celer, T. Kowalewski, M. Jaroniec, K. Matyjaszewski, Grafting monodisperse polymer chains from concave surfaces of ordered mesoporous silicas. *Macromolecules* **41**, 8584–8591 (2008).

43. U. M. Ingle, A. M. Lali, Significance of porosity and pore accessibility for the selection of ion exchange adsorbents for chromatographic purification of macromolecules. *Acta Chromatogr.* **29**, 5–24 (2017).

44. S. Brandt, R. A. Goffe, S. B. Kessler, J. L. O'Connor, S. E. Zale, Membrane-based affinity technology for commercial scale purifications. *Nat. Biotechnol.* **6**, 779–782 (1988).

45. C. Boi, A. Malavasi, R. G. Carbonell, G. Gilleskie, A direct comparison between membrane adsorber and packed column chromatography performance. *J. Chromatogr. A* **1612**, 460629 (2020).

46. C. Boi, Membrane adsorbers as purification tools for monoclonal antibody purification. *J. Chromatogr. B* **848**, 19–27 (2007).

47. A. C. A. Roque, A. S. Pina, A. M. Azevedo, R. Aires-Barros, A. Jungbauer, G. Di Profio, J. Y. Y. Heng, J. Haigh, M. Ottens, Anything but conventional chromatography approaches in bioseparation. *Biotechnol. J.* **15**, e1900274 (2020).

48. J. N. Mabry, M. J. Skaug, D. K. Schwartz, Single-molecule insights into retention at a reversed-phase chromatographic interface. *Anal. Chem.* **86**, 9451–9458 (2014).

49. N. A. Moringo, L. D. C. Bishop, H. Shen, A. Misiura, N. C. Carrejo, R. Baiyasi, W. Wang, F. Ye, J. T. Robinson, C. F. Landes, A mechanistic examination of salting out in protein–polymer membrane interactions. *Proc. Natl. Acad. Sci. U.S.A.* **116**, 22938–22945 (2019).

50. A. Misiura, H. Shen, L. Tauzin, C. Dutta, L. D. C. Bishop, N. C. Carrejo, J. Zepeda O, S. Ramezani, N. A. Moringo, A. B. Marciel, P. J. Rossky, C. F. Landes, Single-molecule dynamics reflect IgG conformational changes associated with ion-exchange chromatography. *Anal. Chem.* **93**, 11200–11207 (2021).

51. L. D. C. Bishop, C. F. Landes, From a protein's perspective: Elution at the single-molecule level. *Acc. Chem. Res.* **51**, 2247–2254 (2018).

52. J. J. van Deemter, A. Klinkenberg, F. J. Zuiderweg, Longitudinal diffusion and resistance to mass transfer as causes of nonideality in chromatography. *Chem. Eng. Sci.* **5**, 271–289 (1956).

53. J. W. M. Osterrieth, J. Rampersad, D. Madden, N. Rampal, L. Skoric, B. Connolly, M. D. Allendorf, V. Stavila, J. L. Snider, R. Ameloot, J. Marreiros, C. Ania, D. Azevedo, E. Vilarrasa-Garcia, B. F. Santos, X.-H. Bu, Z. Chang, H. Bunzen, N. R. Champness, S. L. Griffin, B. Chen, R.-B. Lin, B. Coasne, S. Cohen, J. C. Moreton, Y. J. Colón, L. Chen, R. Clowes, F.-X. Coudert, Y. Cui, B. Hou, D. M. D'Alessandro, P. W. Doheny, M. Dincă, C. Sun, C. Doonan, M. T. Huxley, J. D. Evans, P. Falcaro, R. Ricco, O. Farha, K. B. Idrees, T. Islamoglu, P. Feng, H. Yang, R. S. Forgan, D. Bara, S. Furukawa, E. Sanchez, J. Gascon, S. Telalović, S. K. Ghosh, S. Mukherjee, M. R. Hill, M. M. Sadiq, P. Horcajada, P. Salcedo-Abraira, K. Kaneko, R. Kukobat, J. Kenvin, S. Keskin, S. Kitagawa, K.-I. Otake, R. P. Lively, S. J. A. De Witt, P. Llewellyn, B. V. Lotsch, S. T. Emmerling, A. M. Pütz, C. Martí-Gastaldo, N. M. Padial, J. García-Martínez, N. Linares, D. Maspoch, J. A. Suárez Del Pino, P. Moghadam, R. Oktavian, R. E. Morris, P. S. Wheatley, J. Navarro, C. Petit, D. Danaci, M. J. Rosseinsky, A. P. Katsoulidis, M. Schröder, X. Han, S. Yang, C. Serre, G. Mouchaham, D. S. Sholl, R. Thyagarajan, D. Siderius, R. Q. Snurr, R. B. Goncalves, S. Telfer, S. J. Lee, V. P. Ting, J. L. Rowlandson, T. Uemura, T. Iiyuka, M. A. van der Veen, D. Rega, V. Van Speybroeck, S. M. J. Rogge, A. Lamaire, K. S. Walton, L. W.

Bingel, S. Wuttke, J. Andreo, O. Yaghi, B. Zhang, C. T. Yavuz, T. S. Nguyen, F. Zamora, C. Montoro, H. Zhou, A. Kirchon, D. Fairen-Jimenez, How reproducible are surface areas calculated from the BET equation? *Adv. Mater.* **34**, e2201502 (2022).

54. P. DePhillips, A. M. Lenhoff, Pore size distributions of cation-exchange adsorbents determined by inverse size-exclusion chromatography. *J. Chromatogr. A* **883**, 39–54 (2000).

55. J. P. Hanrahan, SOLAS underivatized silica certificate of analysis (Glantreo, 2022).

56. H. L. Sung, D. J. Nesbitt, Ligand-dependent volumetric characterization of manganese riboswitch folding: A high-pressure single-molecule kinetic study. *J. Phys. Chem. B* **126**, 9781–9789 (2022).

57. J. S. H. Danial, J. Y. L. Lam, Y. Wu, M. Woolley, E. Dimou, M. R. Cheetham, D. Emin, D. Klenerman, Constructing a cost-efficient, high-throughput and high-quality single-molecule localization microscope for super-resolution imaging. *Nat. Protoc.* **1711**, 2570–2619 (2022).

58. M. N. Alsamsam, A. Kopūstas, M. Jurevičiūtė, M. Tutkus, The miEye: Bench-top super-resolution microscope with cost-effective equipment. *HardwareX* **12**, e00368 (2022).

59. S. N. Kramer, J. Antarasen, C. R. Reinholt, L. Kisley, A practical guide to light-sheet microscopy for nanoscale imaging: Looking beyond the cell. *J. Appl. Phys.* **136**, 091101 (2024).

60. J. Chen, A. Bremauntz, L. Kisley, B. Shuang, C. F. Landes, KisleyLabAtCWRU/SuperResKinetics, version 20231026, Zenodo (2023); https://zenodo.org/records/10048387.

61. L. Li, B. O. Xin, W. Kuang, Z. Zhou, Z.-L. Huang, Divide and conquer: Real-time maximum likelihood fitting of multiple emitters for super-resolution localization microscopy. *Opt. Express* **27**, 21029–21049 (2019).

62. D. Sage, T.-A. Pham, S. Holden, Super-resolution fight club: Assessment of 2D and 3D single-molecule localization microscopy software. *Nat. Methods* **16**, 387–395 (2019).

63. D. Sage, H. Kirshner, T. Pengo, N. Stuurman, J. Min, S. Manley, M. Unser, Quantitative evaluation of software packages for single-molecule localization microscopy. *Nat. Methods* **12**, 717–724 (2015).

64. K. J. A. Martens, A. N. Bader, S. Baas, B. Rieger, J. Hohlbein, Phasor based single-molecule localization microscopy in 3D (pSMLM-3D): An algorithm for MHz localization rates using standard CPUs. *J. Chem. Phys.* **148**, 123311 (2018).

65. A. C. Borges-Muñoz, D. P. Miller, E. Zurek, L. A. Colón, Silanization of superficially porous silica particles with p-aminophenyltrimethoxysilane. *Microchem. J.* **147**, 263–268 (2019).

66. N. Singh, J. Wang, M. Ulbricht, S. R. Wickramasinghe, S. M. Husson, Surface-initiated atom transfer radical polymerization: A new method for preparation of polymeric membrane adsorbers. *J. Memb. Sci.* **309**, 64–72 (2008).

67. J. Chen, A. Bremauntz, L. Kisley, B. Shuang, C. F. Landes, KisleyLabAtCWRU/SuperResKinetics: Super-resolution imaging and single molecule kinetics MATLAB analysis, GitHub (2013); https://github.com/KisleyLabAtCWRU/SuperResKinetics.

68. R. Parthasarathy, Rapid, accurate particle tracking by calculation of radial symmetry centers. *Nat. Methods* **97**, 724–726 (2012).

69. O. Woodford, vol3d v2, MathWorks (2022); www.mathworks.com/matlabcentral/fileexchange/22940-vol3d-v2.

70. S. D. Verma, D. A. Vanden Bout, M. A. Berg, When is a single molecule heterogeneous? A multidimensional answer and its application to dynamics near the glass transition. *J. Chem. Phys.* **143**, 024110 (2015).

71. L. Pasti, A. Cavazzini, M. Nassi, F. Dondi, Dynamic chromatography: A stochastic approach. *J. Chromatogr. A* **1217**, 1000–1009 (2010).

72. M. Tokunaga, N. Imamoto, K. Sakata-Sogawa, Highly inclined thin illumination enables clear single-molecule imaging in cells. *Nat. Methods* **52**, 159–161 (2008).

73. O. Gal, fit_ellipse. MATLAB Central File Exchage, MathWorks (2023); www.mathworks.com/matlabcentral/fileexchange/3215-fit_ellipse.

74. M. Thommes, K. Kaneko, A. V Neimark, J. P. Olivier, F. Rodriguez-Reinoso, J. Rouquerol, K. S. W. Sing, Physisorption of gases, with special reference to the evaluation of surface area and pore size distribution (IUPAC Technical Report). *Pure Appl. Chem.* **87**, 1051–1069 (2015).

75. S. Brunauer, P. H. Emmett, E. Teller, Adsorption of gases in multimolecular layers. *J. Am. Chem. Soc.* **60**, 309–319 (1938).

76. J. Rouquerol, F. Rouquerol, P. Llewellyn, G. Maurin, K. Sing, *Adsorption by Powders and Porous Solids: Principles, Methodology and Applications* (Academic Press, 2013).

77. M. D. Abramoff, P. J. Magalhães, S. J. Ram, Image processing with ImageJ. *Biophotonics Int.* **11**, 36–42 (2004).

78. Regis Technologies, Reflect Coated Care & Use Guide (Regis Technologies, 2023).

79. E. G. Franklin, M. Wilcox, S. Anderson, "Sample loadability on coated and immobilized polysaccharide-based CSPs" (Regis Technologies Inc.).

80. Belay A, Assefa G, Concentration, wavelength and temperature dependent refractive index of sugar solutions and methods of determination contents of sugar in soft drink beverages using laser lights. *J. Lasers Opt. Photonics* **05**, 1–5 (2018).

81. J. Agustian, A. Harun Kamaruddin, H. Y. Aboul-enein, Chromatographic comparison of atenolol separation in reaction media on cellulose tris-(3,5-dimethylphenylcarbamate) chiral stationary phase using ultra fast liquid chromatography. *Chirality* **24**, 356–367 (2012).

82. R. Walder, M. Kastantin, D. K. Schwartz, High throughput single molecule tracking for analysis of rare populations and events. *Analyst* **137**, 2987–2996 (2012).

83. L. Kisley, J. Chen, A. P. Mansur, S. Dominguez-Medina, E. Kulla, M. K. Kang, B. Shuang, K. Kourentzi, M. V. Poongavanam, S. Dhamane, R. C. Willson, C. F. Landes, High ionic strength

narrows the population of sites participating in protein ion-exchange adsorption: A single-molecule study. *J. Chromatogr. A* **1343**, 135–142 (2014).

84. Y. Liu, X. Wang, J. Yu, X. Guo, Chiral separation and molecular simulation study of six antihistamine agents on a coated cellulose tri-(3,5-dimethylphenycarbamate) column (Chiralcel OD-RH) and its recognition mechanisms. *Electrophoresis* **42**, 1461–1472 (2021).
